# Global Diversity of Sponges (Porifera)

**DOI:** 10.1371/journal.pone.0035105

**Published:** 2012-04-27

**Authors:** Rob W. M. Van Soest, Nicole Boury-Esnault, Jean Vacelet, Martin Dohrmann, Dirk Erpenbeck, Nicole J. De Voogd, Nadiezhda Santodomingo, Bart Vanhoorne, Michelle Kelly, John N. A. Hooper

**Affiliations:** 1 Netherlands Centre for Biodiversity Naturalis, Leiden, The Netherlands; 2 Aix-Marseille University, Centre d'Océanologie de Marseille, CNRS, DIMAR, UMR 6540, Marseille, France; 3 Department of Invertebrate Zoology, National Museum of Natural History, Smithsonian Institution, Washington, D.C., United States of America; 4 Department of Earth- and Environmental Sciences & GeoBio-Center LMU, Ludwig-Maximilians-University Munich, Munich, Germany; 5 Paleontology Department, Natural History Museum, London, United Kingdom; 6 Flanders Marine Institute - VLIZ, Innovocean Site, Oostende, Belgium; 7 National Centre for Aquatic Biodiversity & Biosecurity, National Institute of Water and Atmospheric Research Ltd, Auckland, New Zealand; 8 Queensland Museum, South Brisbane, Queensland, and Eskitis Institute for Cell & Molecular Therapies, Griffiths University, Queensland, Australia; Heriot-Watt University, United Kingdom

## Abstract

With the completion of a single unified classification, the Systema Porifera (SP) and subsequent development of an online species database, the World Porifera Database (WPD), we are now equipped to provide a first comprehensive picture of the global biodiversity of the Porifera. An introductory overview of the four classes of the Porifera is followed by a description of the structure of our main source of data for this paper, the WPD. From this we extracted numbers of all ‘known’ sponges to date: the number of valid Recent sponges is established at 8,553, with the vast majority, 83%, belonging to the class Demospongiae. We also mapped for the first time the species richness of a comprehensive set of marine ecoregions of the world, data also extracted from the WPD. Perhaps not surprisingly, these distributions appear to show a strong bias towards collection and taxonomy efforts. Only when species richness is accumulated into large marine realms does a pattern emerge that is also recognized in many other marine animal groups: high numbers in tropical regions, lesser numbers in the colder parts of the world oceans. Preliminary similarity analysis of a matrix of species and marine ecoregions extracted from the WPD failed to yield a consistent hierarchical pattern of ecoregions into marine provinces. Global sponge diversity information is mostly generated in regional projects and resources: results obtained demonstrate that regional approaches to analytical biogeography are at present more likely to achieve insights into the biogeographic history of sponges than a global perspective, which appears currently too ambitious. We also review information on invasive sponges that might well have some influence on distribution patterns of the future.

## Introduction

**Figure 1:**
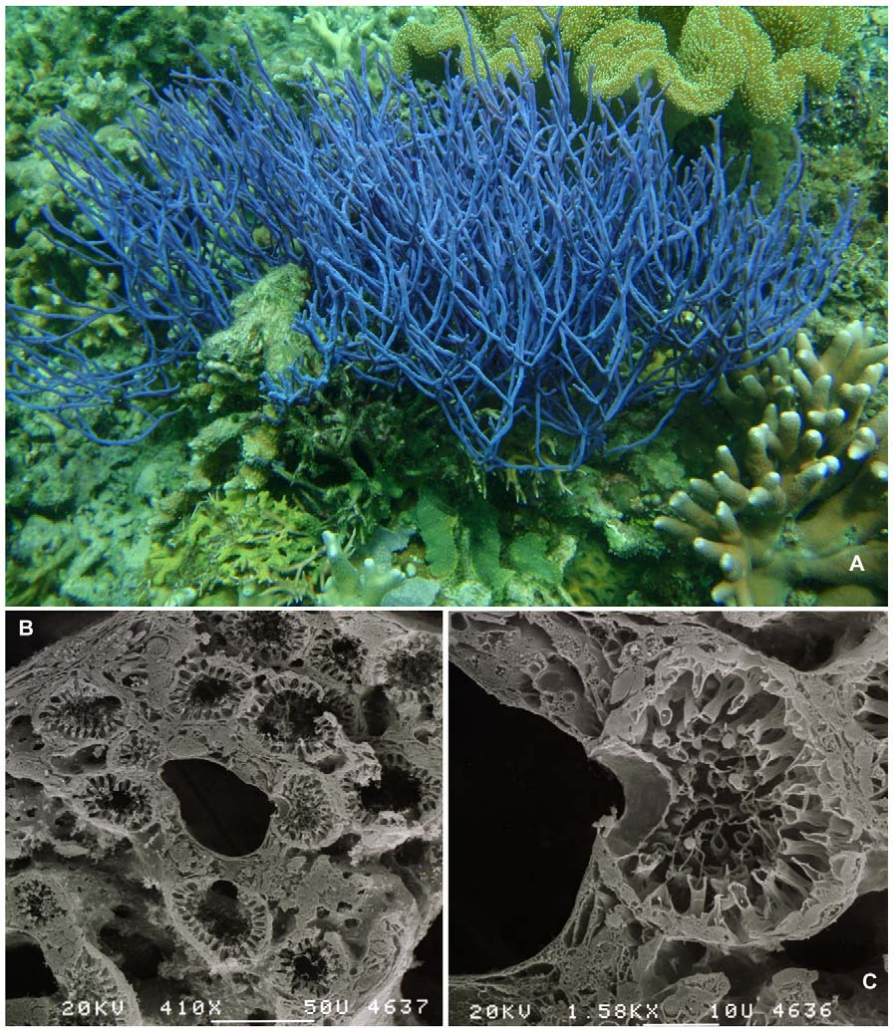
Porifera morphology and internal structure. A. *Callyspongia (Callyspongia) samarensis* (Demospongiae: Haplosclerida), Ternate, Maluku province, Indonesia (photo N.J. de Voogd); B. SEM image of cross section of mesohyl of the demosponge *Scopalina ruetzleri* obtained by freeze-fracturing technique (courtesy L. de Vos); C. Detail of choanocyte chamber of *Scopalina ruetzleri* (courtesy L. de Vos).

Sponges, phylum Porifera, are the oldest metazoan group still extant on our planet. Their continued survival in vast numbers in Recent seas (and in freshwater habitats) is closely linked to the apparent adaptability of their bauplan to dramatic changes in environmental characteristics and competing biota [1], [2]. Sponges (Fig. 1A) are exclusively aquatic animals, which are fixed on the substrate and live by drawing in water and filtering microscopic-size food particles from it. Recent research also indicates an ability to take up dissolved organic matter [3]. Sponges have a simple level of organization: there are specialized cells for a variety of life functions, but these are not organized into tissues or organs. All sponges have a “skin” of T-shaped or flattened cells (called pinacocytes) which covers the outside of the sponge) as well as its internal system of canals, and microscopic chambers (Fig. 1B). These chambers have a lining of flagella-bearing cells (choanocytes, Fig. 1C) that generate the water currents necessary for the unique filtering activity characteristic to sponges. An exception to this is in the so-called carnivorous sponges, highly adapted deep-sea forms, in which the aquiferous system is non-existent, but which have a sticky outer surface with which small prey animals are captured [4]. The space (Fig. 1B) between canals and chambers is filled with a collagenous matrix, called the mesohyl, which harbors individual cells, supporting fibers, and inorganic structures of the skeleton [5].

Sponges grow in distinct shapes (Fig. 1A) and sizes due to the form of the internal mineral and/or organic skeletons secreted by specialized cells. The skeleton may also be supplemented by exogenous materials, such as sand grains. Skeletons, when present, are constructed of discrete siliceous or calcareous elements (*spicules*) and/or organic collagenous fibers (*spongin*), and rarely skeletons may be aspicular massive limestone constructions. Depending on the nature and density of these building components, sponge species may variously be soft, compressible, fragile or rock hard in consistency. Sponges come in various shapes and sizes, from flat cushions to elaborate branching or cup-shaped forms, from tiny crusts measured in mm, to giant shapes in meters. Sponges have numerous microscopic openings (the incurrent pores) and one or a few larger vents (the excurrent oscules). The shapes of sponges are variable among different species and genera, but also vary to some extent between individuals of the same species in response to environmental factors such as hydrodynamics, light and turbidity.

A great diversity of symbiotic organisms often thrive inside or on the body of a sponge, from microscopic prokaryotes, e.g. [6], [7] to macroscopic organisms such as shrimps, polychaetes, hydrozoans and fishes, e.g. [8].

The simple body organization of sponges and relative plasticity of the cellular elements, coupled with a unique tolerance towards symbiotic microorganisms, allows for a great diversity of ‘evolutionary solutions’ for environmental challenges. Knowledge of sponge biodiversity is still largely incomplete. To date, about 11,000 species have been formally described of which approximately 8,500 are considered valid (see below), but as many as twice that number are thought to exist. Sponges are currently divided among four distinct classes, 25 orders, 128 families and 680 genera [9], [10], but many of these higher taxa are under discussion due to new insights obtained from molecular systematic methods and new considerations of their morphological characteristics. Fossil sponges comprise a similar additional diversity [11] There are several hundred freshwater species.

Due to the limited swimming capabilities of most sponge larvae, and occasional asexual propagation, most sponges occur in regional or local areas of endemism, unless spread globally or regionally in an inadvertent manner by shipping traffic. Sponges may be found vertically from the eulittoral zone to hadal depths, horizontally from the tropics to the highest latitudes, locally from epifaunal rocky communities to mud bottoms and ephemeral freshwater habitats. Their importance for the global ecosystem is high but not widely appreciated [12], [13]. Sponges are efficient filter feeders, vital to the health and economics of all marine systems by linking the nutrients of the open water column with benthic communities. Symbionts of sponges play a decisive role in the nitrogen cycle of many habitats and may contribute significantly to organic production in oligotrophic habitats. Specialized sponges are important bio-eroders in coral reefs, coralline bottoms and oyster beds and they may compete successfully with other sessile organisms such as corals. Specific groups have an essential function in binding unconsolidated substrate such as coral rubble and pebbles into stable surfaces. Many fossil sponges and a small group of Recent sponges are capable of building extensive reef formations that today, in some locations, shape the contours of the benthos, and now form uplifted terrestrial habitats. Megabenthic species may form high-density aggregations in many shelf edge and seamount regions playing a so far unexplored role in deep-sea ecosystems. These are only a few general features of the ecosystem services provided by the global sponge community [14]–[26].

Although sponges have been known to mankind since the earliest civilizations (4000 YBP, see [27]) they were not recognized as an independent metazoan lineage until well into the 19^th^ century, when Robert Grant [28] first observed their unique morphology and physiology and coined the name Porifera for them. Since then, spongology, the study of all aspects of the biology, ecology, taxonomy and chemistry of sponges, has grown into a discipline attracting a steadily increasing population of hundreds of scientists worldwide, many of whom devote a lifetime career to the study of this group. Increasingly, sponges are studied as part of a broader enterprise attempting to detail the Tree of Life. Apart from nurturing academic interest, sponges play an important role in human health as producers of chemical compounds with useful pharmaceutical properties, including antitumor, anti-infective and anti-inflammatory properties [29]. Natural sponges are still harvested for personal, industrial, and artistic use.

For the first time since the appearance of the 2002 consensus classification, we review here the global diversity of the Recent Porifera, giving a summary of the major groups and their currently established taxon richness. We also make a first attempt to review distribution patterns of species and higher taxa over the global seas and oceans. Our emphasis will be on the ‘known’ species, but we will also briefly consider the ‘unknown’ species.

## Methods

Because of the review nature of this study, methods employed are diverse. We summarize here the major methodological approaches, which are further explained in the various sections below. Taxonomic and distribution data were extracted from the online World Porifera Database [10] (accessed 2011 Sept 30), and supplemented with a survey of the literature on sponge diversity. Figures, tables and maps are partially the result of newly analyzed data. The type localities and additional confirmed occurrences in neighboring areas of almost all ‘accepted’ species were entered in the WPD in generalized areas (Marine Ecoregions of the World, MEOWs, see [30]), but many non-original distribution records are still to be evaluated and entered. Moreover, many sponge taxa are recorded in the literature as ‘undetermined’ and these are not included in the WPD. Thus, the data and maps for species presented here are to be considered a conservative or ‘minimal’ estimate of the actual distributional data and patterns. For the production of maps and the tracing of species richness patterns, WPD data sets were combined in geographic information system (GIS) software (ESRI ArcGIS v9.3). A biodiversity analysis aimed at testing the aptness of the MEOW hierarchical system of Marine Provinces and Marine Realms for sponge richness data was carried out using the Bray-Curtis coefficient hierarchical clustering of WPD datasets performed with the PRIMER-6 (PRIMER-E) package. Presence/absence sponge species data were clustered at three levels distinguished in the MEOW [30] system: realm, province (>50 records) and ecoregion (>20 records) level. The reduction in the number of provinces and ecoregions was determined empirically by repeated clustering attempts with different minimum record numbers in which level of resolution of the dendrogram was observed. This reduction is justified by the lack of sufficient exploration of these geographic units, but precise levels (minimum of 50 and 20 records) were chosen arbitrarily. Author contributions outlined below were solicited on the basis of expert knowledge and skills.

## Results

### Currently recognized higher taxa and new (molecular) developments

#### Demospongiae

**Figure 2:**
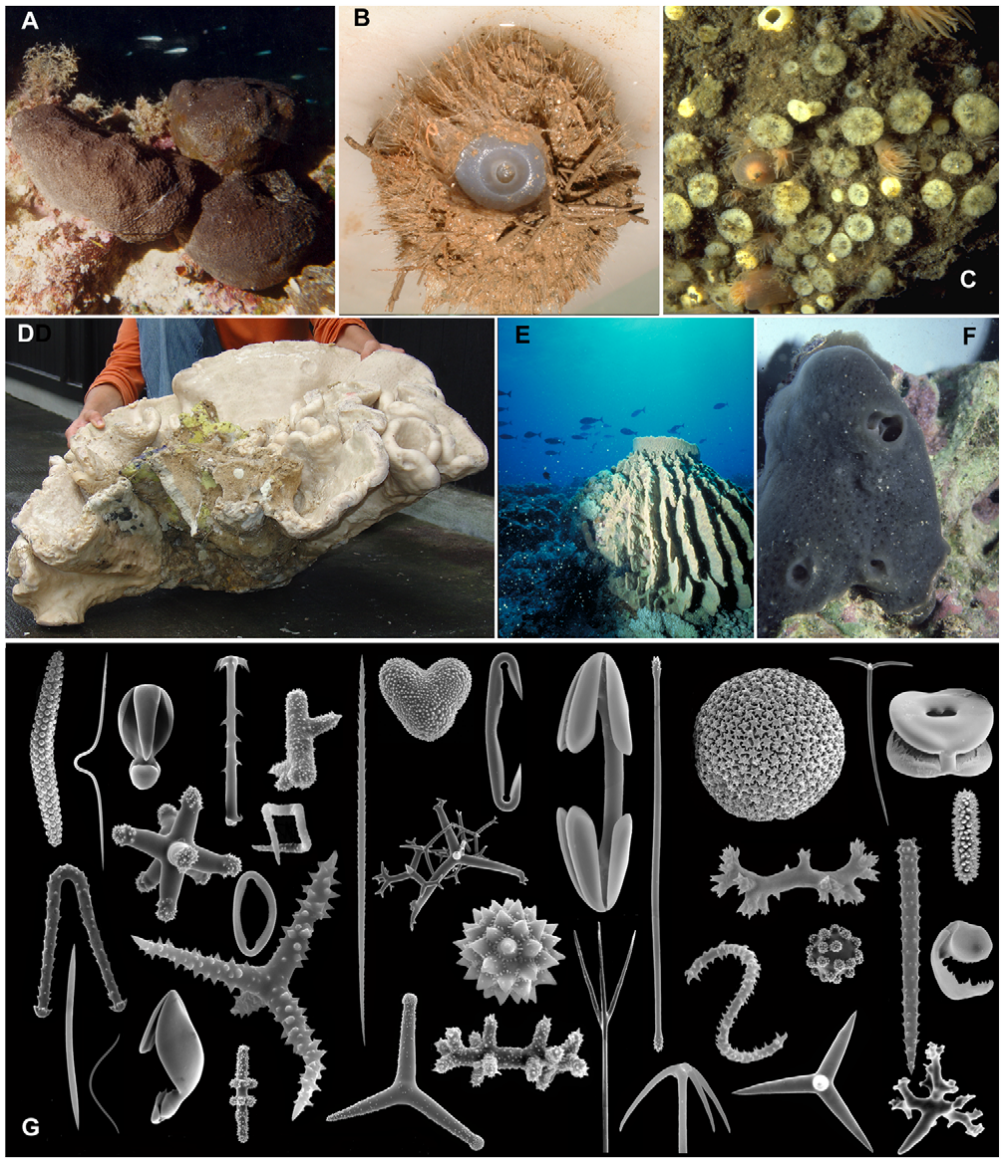
Demospongiae morphology and spicule diversity. A. Bath sponge, *Spongia officinalis*, Greece (photo courtesy E. Voultsiadou); B. Bathyal mud sponge *Thenea schmidti*; C. Papillae of excavating sponge *Cliona celata* protruding from limestone substratum (photo M.J. de Kluijver); D. Giant rock sponge, *Neophrissospongia*, Azores (photo F.M. Porteiro/ImagDOP); E. Giant barrel sponge *Xestospongia testudinaria*, Lesser Sunda Islands, Indonesia (photo R. Roozendaal); F. *Amphimedon queenslandica* (photo of holotype in aquarium, photo S. Walker); G. SEM images of a selection of microscleres and megascleres, not to scale, sizes vary between 0.01 and 1 mm.

Demospongiae is the largest and most diverse class of the Porifera. It unites [9] sponges with siliceous spicules (Fig. 2G) (either monaxonic or tetraxonic, never triaxonic) and/or with a skeleton of organic fibers or fibrillar collagen. Like in Hexactinellida (see below) siliceous spicules are divided into megascleres, which strengthen the framework of the sponges, and microscleres, which have various – possibly defensive, possibly supportive of soft tissues, but generally unclear – functions. Microscleres are frequently more common in the outer regions of the sponges and often surround aquiferous canals. Members of the class Homoscleromorpha also possess tetraxonic siliceous spicules, but they lack a subdivision in mega- and microscleres. Occasionally the skeleton is absent, a feature shared again with some Homoscleromorpha. Rare forms with limestone basal skeletons are living links to Paleozoic reef-building sponges. Larvae are usually of the parenchymella type (solid with overall ciliation), but in some groups hollow larvae occur [31], [32]. The most recent summary of the Porifera classification [9] recognized 15 ordinal groups, one of which was recently transferred to the class Homoscleromorpha (see below). The major groups include three orders possessing tetraxonic spicules (Spirophorida, Astrophorida, and part of the “Lithistida”), three orders lacking siliceous spicules that were historically called keratose or horny sponges (Dictyoceratida, Dendroceratida, and Verongida), a single large order based on the possession ‘chelae’ microscleres (order Poecilosclerida) and a single large order based on the possession of skeletons built in a reticulate arrangement of simple diactinal spicules called ‘oxeas’ and ‘strongyles’ (order Haplosclerida). Freshwater sponges have so far been included in the latter order, but are probably unrelated (see below). There are also several less firmly established orders that are based upon unique combinations of non-exclusive skeletal or spicule characters (orders Hadromerida, Halichondrida), or smaller groups with unique skeletal or spicule features (Agelasida, Chondrosida+Halisarcida). The integrity of these groups is currently being investigated using molecular techniques and proposals to rearrange all ordinal groups and their families is imminent ([33]; see also below).

Demosponges demonstrate a tremendous diversity that can only be illustrated with a few iconic examples: The well-known bath sponges (family Spongiidae, Fig. 2A) have excellent properties to appeal to human use as a cleaning or scrubbing tool: a softly compressible consistency and a silica-free resilient skeleton of horny fibres. They grow in warmer waters worldwide and have been exploited to near-extinction in many areas. Nowadays, use of bath sponges is limited to specialized industries and as a curiosity for tourists [34]. Deep-sea species of the genus *Thenea* (Astrophorida, Fig. 2B), have strongly differentiated hairy stalked bodies specialized in living on bathyal and abyssal mud flats, using long laterally spreading spicules and basal roots. Excavating (or boring) sponges (Fig. 2C) are able to penetrate and erode limestone surfaces. They belong to families Clionaidae (order Hadromerida), Thoosidae (order Astrophorida) and genus *Aka* (family Phloeodictyidae). The sponges use acid produced by special cells to etch small ‘chips’ of calcium carbonate [35] from the substratum and through this activity recycle limestone in e.g. coral reef ecosystems, coralline bottoms and temperate oysterbanks. Rock sponges, “Lithistida” (Fig. 2D), are a polyphyletic group of sponges with stone-hard silica skeletons composed of intimately interlocking spicules. Many living species are found in deeper waters of tropical and (warm-)temperate regions and are thought to be isolated survivors of a much larger fossil sponge fauna, e.g. [36]. ‘Giant barrel sponges’, e.g. the haplosclerid *Xestospongia muta*, referred to by some as ‘Redwoods of the Reef’ [37], [38], have been estimated to reach ages of 2000 years or more in Caribbean seas. A counterpart species in the Indo-Pacific (*X. testudinaria*, Fig. 2E) shows comparable sizes and may be similarly long-lived. The Australian haplosclerid *Amphimedon queenslandica* (Fig. 2F) was the first, and thus far only sponge to have its entire genome sequenced [39], [40]. It proved beyond reasonable doubt that sponges are at the very base of the Metazoan Tree of Life.

#### Carnivorous sponges

Some sponges of the order Poecilosclerida, class Demospongiae, have a surprising carnivorous feeding regime [4], [41], [42], instead of being filter-feeders, as is typical of sponges. These typically deep-sea sponges lack the aquiferous system and the choanocyte cells which are considered to be diagnostic for Porifera [1]. Most display a peculiar symmetrical shape, generally with lateral appendages lined by hook-like microsclere spicules forming a sticky ‘velcro’-like cover on which prey are trapped. An aquiferous system is maintained only in the genus *Chondrocladia*, in which, however, it is apparently not used for water filtration but for the inflation of turgescent spheres lined by the same sticky cover of hook-like spicules. They prey on a variety of small invertebrates, mostly crustaceans, with setae or bristles that ensnare on the spicule cover. In the absence of any gut or digestive cavity, digestion is performed by cells migrating toward the prey and acting individually to phagocytize and digest its fragments intracellularly [43]. This system is unique in the Metazoa, but it parallels the behaviour of individual sponge cells, which perform the various functions of differentiated tissue, organs and a nervous system, which sponges lack.

By the end of the twentieth century, 90 carnivorous sponges were classified in the family Cladorhizidae, within three genera, *Cladorhiza*, *Asbestopluma* and *Chondrocladia*. They were all found in the deep sea, including the depth record for sponges, with a species known from 8840 m. Increased interest in these sponges, due to the discovery that they are carnivorous, and due to the development of manned submersibles and ROVs, has shown that this diversity was largely underestimated. To date, 119 species are known, classified in three families and eight genera, and several new species and a new genus are in the course of description.

**Figure 3:**
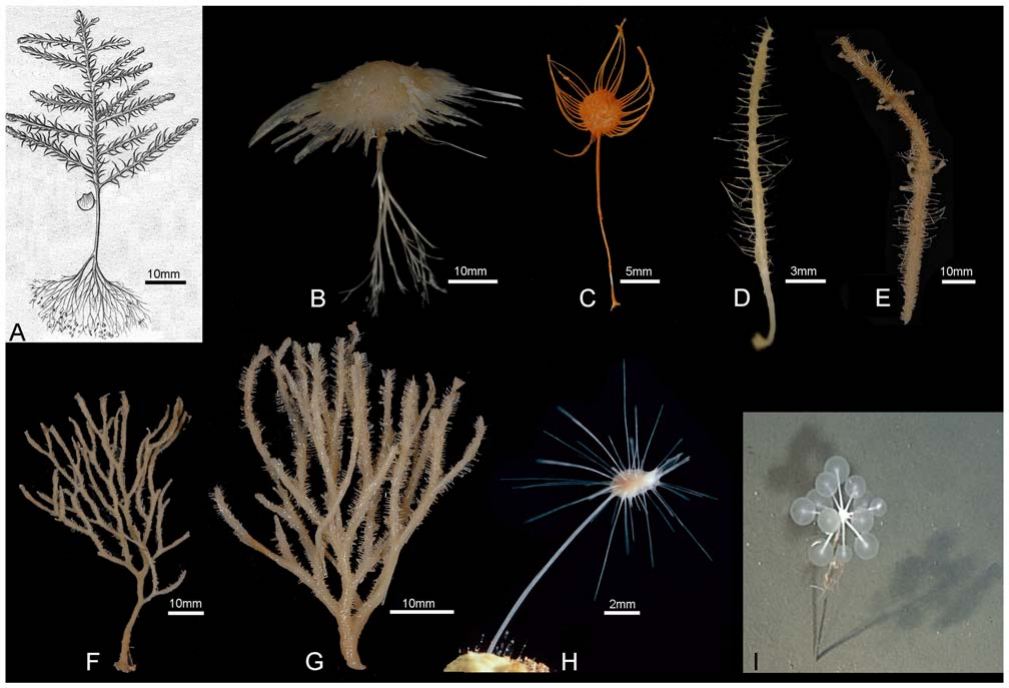
Carnivorous sponge diversity. A. *Cladorhiza abyssicola* (from Fig. 17 in [172], scale approximate); B. *Cladorhiza* sp., undescribed species from West Norfolk Ridge (New Zealand EEZ), 757 m (NIWA 25834); C. *Abyssocladia* sp., undescribed species from Brothers Seamount (New Zealand EEZ), 1336 m (NIWA 21378); D. *Abyssocladia* sp., undescribed species from Chatham Rise (New Zealand EEZ), 1000 m (NIWA 21337); E. *Abyssocladia* sp., undescribed species from Seamount 7, Macquarie Ridge (Australian EEZ), 770 m (NIWA 40540); F. *Asbestopluma (Asbestopluma) desmophora*, holotype QM G331844, from Macquarie Ridge (Australian EEZ), 790 m (from Fig. 5A in [47]); G. *Abyssocladia* sp., undescribed species from Seamount 8, Macquarie Ridge (Australian EEZ), 501 m (NIWA 52670); H. *Asbestopluma hypogea* from [41]; I. *Chondrocladia lampadiglobus* (from Fig. 17A in [48]).

The morphology of carnivorous sponges is always erect, but is highly diverse (Fig. 3) and often poorly known because they are fragile and easily broken during collection in dredges. Their stalk may be attached to hard substrate by an enlarged base or rooted in the sediment. Some are feather-shaped, others are pedunculate with a disc-shaped body bearing radiating filaments, while others have a fan-shaped morphology which may be confused with that of hydroids or gorgonians. Some *Chondrocladia* spp. are stalked, with lateral processes ending in translucent inflated spheres.

The evidence that this special morphology is related to a carnivorous habit has been first obtained in an *Asbestopluma* species living in a cool-water littoral cave [4]. The latter species was able to thrive in laboratory conditions, offering excellent study conditions [43]. Although a carnivorous regime is difficult to prove conclusively in the deep sea, it appears likely since several deep-sea sponges sharing this morphology have shown partially digested crustaceans included in their body, see [44], [45].

The spicule skeleton, on which the classification is based, includes monaxonic megascleres that usually include a special type of tylostyle, a mycalostyle, that builds the axes of the body and of the appendages, and a large variety of microscleres, generally chelae and derivatives, to which may be added sigmas, sigmancistras, microstyles and forceps. Interestingly, the chelae microscleres have no known function in other poecilosclerids, but in carnivorous species seem to be used to trap the prey, by lining the surface of body and appendages with the larger hook outwardly directed.

**Figure 4:**
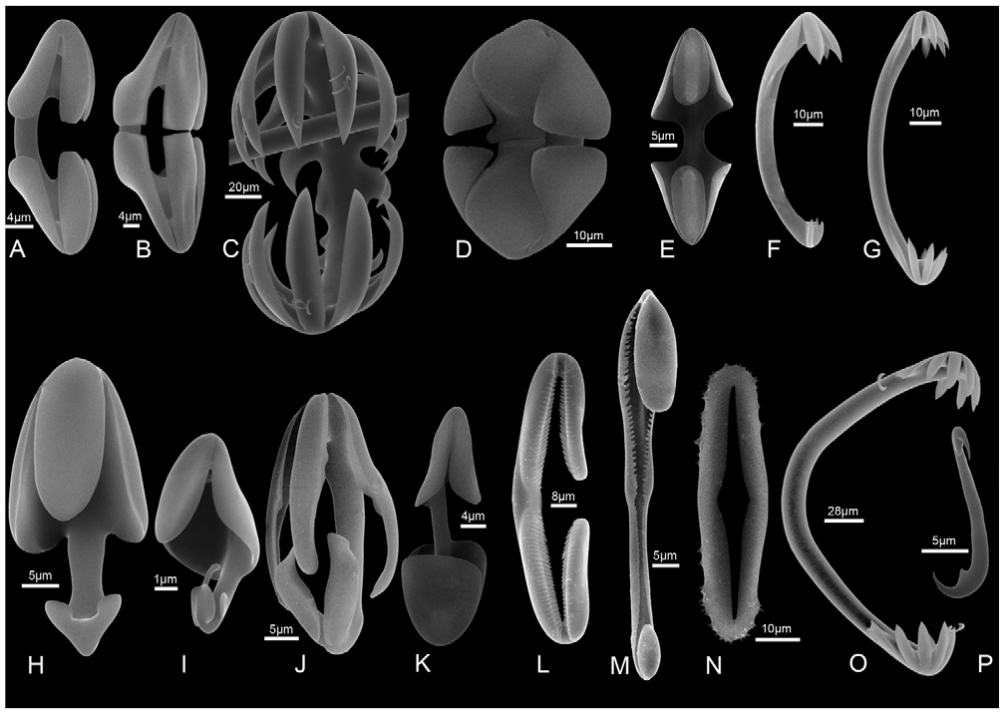
Examples of chelae and sigmancistras in carnivorous sponges. A. Arcuate isochelae from *Abyssocladia* sp., an undescribed species from Morgue Seamount, Chatham Rise (New Zealand EEZ), 1000 m (NIWA 21337); B. Abyssochela from *Abyssocladia* sp., an undescribed species from Morgue Seamount, Chatham Rise (New Zealand EEZ), 1000 m (NIWA 21337); C. Abyssochela from *Abyssocladia carcharias*, holotype NIWA 62124, from Monowai Seamount, Kermadec Volcanic Arc (New Zealand EEZ, [47]), 1071 m; D. Abyssochela from *Abyssocladia* sp., an undescribed species from Seamount 8, Macquarie Ridge (Australian EEZ), 501 m (NIWA 52670); E. Palmate isochelae from *Abyssocladia* sp. (cf.), an undescribed species from Seamount 7, Macquarie Ridge (Australian EEZ), 770 m (NIWA 40486); F. Anchorate unguiferate anisochela from *Cladorhiza* sp., an undescribed species from West Norfolk Ridge (New Zealand EEZ), 757 m (NIWA 25834); G. Anchorate isochelae from *Chondrocladia (Meliiderma) turbiformis*, holotype NIWA 21357, from Pyre Seamount, Chatham Rise, 1075 m (from Fig. 2D right, in [46]); H–I. Palmate anisochelae from *Asbestopluma* sp., an undescribed species from Hikurangi Channel, off Gisborne, eastern North Island of New Zealand, 1119 m (NIWA 32053); J. Anisochela from *Asbestopluma* sp., an undescribed species from Ghoul Seamount, Chatham Rise, 922 m (NIWA 21343); K. Palmate anisochela from *Abyssocladia* sp., an undescribed species from Seamount 7, Macquarie Ridge (Australian EEZ), 770 m (NIWA 40486); L. Placochela from *Euchelipluma pristina*; M. Anisoplacochela from *Asbestopluma* (*Asbestopluma*) *anisoplacochela*, holotype 25835, from Three Kings Ridge, northern New Zealand, 1690 m [47]; N. Cercichela from *Cercicladia australis*, holotype NIWA 39599, from Seamount 1, Macquarie Ridge, 1060 m, (New Zealand EEZ, [49]) (from Fig. 2H, upper left in [49]); O. Anchorate isochela from *Lollipocladia tiburoni* (from Fig. 3E left, in [50]); P. Sigmancistra from *Asbestopluma* sp., an undescribed species from Hikurangi Channel, off Gisborne, eastern North Island of New Zealand, 1119 m (NIWA 32053).

The diversity of the microscleres is remarkable, especially the apparent derivatives of chelae [46]–[50], in which several new types are known (Fig. 4). These microscleres, although diagnostic of Poecilosclerida, are not in agreement with the sub-ordinal classification of poecilosclerid sponges. The family Cladorhizidae lacks a clear synapomorphy [51], and some sponges with an undoubted carnivorous regime are classified in the families Esperiopsidae or Guitarridae. This could mean either that the classification of Poecilosclerida needs to be revised, or that carnivory appeared before the separation of the evolutionary lineages of Poecilosclerida. Molecular phylogenetic analyses in progress are attempting to resolve this problem.

#### Hexactinellida

Hexactinellida, or glass sponges, are exclusively marine and mainly restricted to hard and soft substrates in deeper waters (200 to >6000 m), although they occasionally occur in shallower, scuba-accessible, water, such as submarine caves in the Mediterranean [52], [53], or off the coast of British Columbia where they form massive structures analogous to Mesozoic sponge reefs, e.g. [54]–[56]. They are mostly inconspicuously coloured and highly variable in body shape (e.g. sac-, vase-, blade-shaped, composed of branching tubes etc.; but never incrusting). Hexactinellids are clearly distinct from other sponges in that their soft tissues are largely syncytial and their siliceous spicules have a triaxonic symmetry; they are viviparous and produce distinctive trichimella larvae (see [57] for a comprehensive review of glass sponge biology). The unusual properties of their spicules have recently attracted the attention of materials scientists, e.g. [58], [59]. Iconic hexactinellids include the venus flower basket (*Euplectella aspergillum*), which often encloses a pair of shrimps inside its body and was used as a bridal gift in ancient Japan, and *Monorhaphis chuni*, which anchors its body in the soft deep-sea floor with a single giant (up to 3 m long) spicule. To date there are ca. 600 described extant species, which is certainly an underestimate of their actual diversity, given their remote habitats and very small number of taxonomic experts for the group [60].

**Figure 5:**
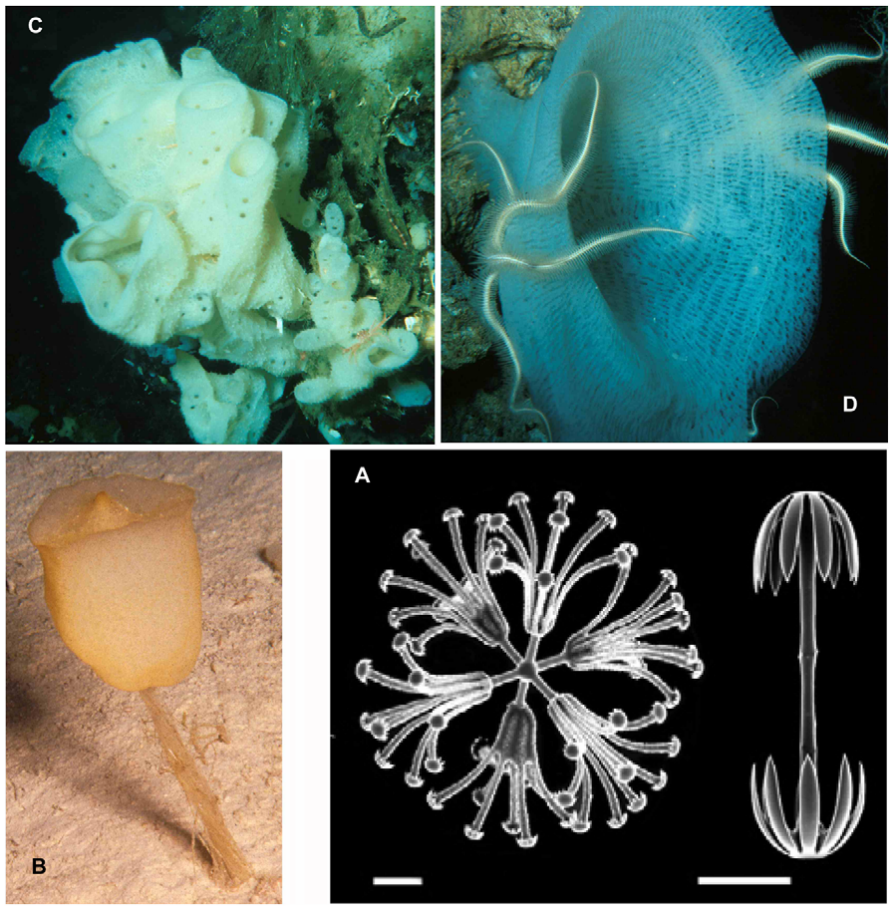
Hexactinellida diversity. A. Scanning electron micrographs of microscleres (courtesy of H.M. Reiswig), left: a hexaster, the diagnostic spicule type of subclass Hexasterophora (scale bar = 10 µm), right: an amphidisc, the diagnostic spicule type of subclass Amphidiscophora (scale bar = 100 µm); B. *Hyalonema* sp., an amphidiscophoran (Amphidiscosida: Hyalonematidae), Bahamas; C. *Atlantisella* sp., a lyssacine hexasterophoran (Lyssacinosida: Euplectellidae), Galapagos Islands; D. *Lefroyella decora*, a dictyonal hexasterophoran (“Hexactinosida”: Sceptrulophora: Euretidae), Bahamas. B–D courtesy of Harbor Branch Oceanographic Institute (Ft. Pierce, Florida, U S A), images taken from manned submersible Johnson-Sea-Link II.

Hexactinellida is divided into two subclasses, the Amphidiscophora, which have amphidisc microscleres, and the Hexasterophora, which have hexaster microscleres (Fig. 5A). Amphidiscophora (Fig. 5B) contains a single extant order with three families; amphidiscophoran species exclusively possess skeletons of unfused spicules. In contrast, Hexasterophora is divided into one order with mostly unfused spicules (Lyssacinosida Fig. 5C with three families) and three orders characterized by fused (dictyonal) main skeletons (Hexactinosida [Fig. 5D] with 9 families, and Aulocalycoida and Lychniscosida with two small families each). Especially the Hexasterophora display an astonishing diversity of spicule forms and skeletal arrangements, and this (for sponges) unusual richness of characters greatly facilitates the delineation of natural taxa. Molecular phylogenetic studies strongly support monophyly of Hexactinellida and its two subclasses, as well as most families and genera sampled so far [61]–[64]. In contrast, order-level phylogeny and classification within Hexasterophora are still poorly resolved since there is strong evidence for paraphyly of Hexactinosida with respect to Lyssacinosida [61] and DNA sequence data for Aulocalycoida, Lychniscosida and many families of Hexactinosida are still missing.

#### Homoscleromorpha

The Homoscleromorpha comprise a small group of marine Porifera with unique features: flagellated opinacocytes and a basement membrane lining both choanoderm and pinacoderm, oval to spherical choanocyte chambers with large choanocytes, and a viviparous cinctoblastula larva. The skeleton, if present, is composed of tetraxonic siliceous spicules with four equal rays (called calthrops) and derivatives showing reduced (diods, triods) or proliferated rays (lophocalthrops). There is no differentiation between megascleres and microscleres, and the spicules are usually small (100 µm or less), not localized in any particular region [65].

**Figure 6:**
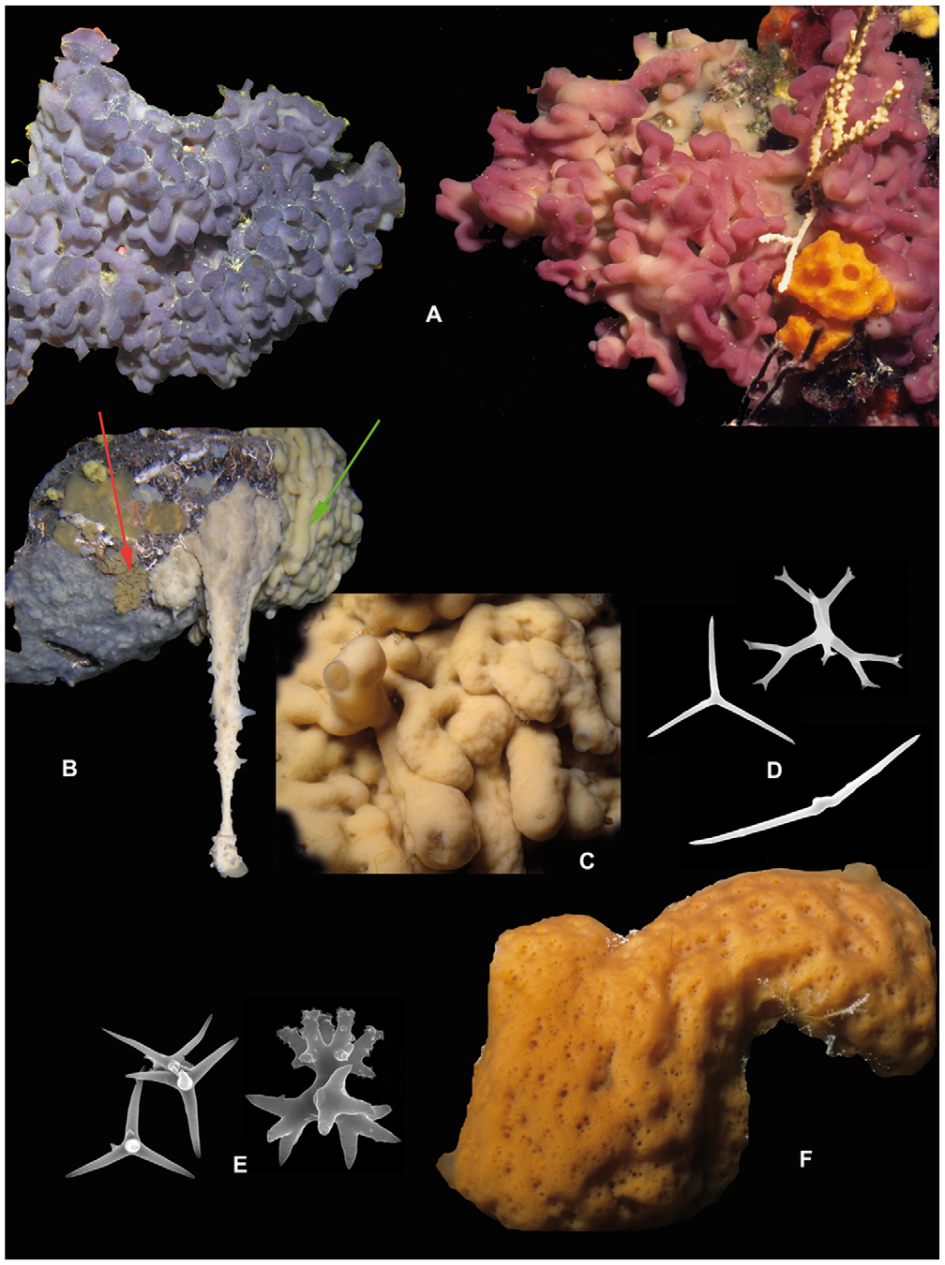
Homoscleromopha diversity. A. *Oscarella lobularis* (Oscarellidae): two color morphs from NW Mediterranean Sea (photos courtesy of Jean Vacelet & Thierry Pérez); B. *Plakortis simplex* (Plakinidae) specimen hanging from the ceiling of the 3PPs cave (NW Mediterranean Sea), a paradise for Homoscleromorpha species (at least 8 species belonging to 4 different genera are present); red arrow indicates the presence of *Oscarella microlobata* and a green arrow *Plakina jani* (photo courtesy Thierry Pérez); C. *Plakina jani* (Plakinidae) detail of the lobes, 3PPs cave (NW Mediterranean Sea) (photo courtesy Jean Vacelet); D. Spicules of Plakinidae: triods, diods and lophose calthrops; E. Spicules of *Corticium candelabrum* (Plakinidae): calthrops and candelabrum (heterolophose calthrops); F. *Corticium candelabrum* NW Mediterranean Sea (photos courtesy of Jean Vacelet).

Most of the species are encrusting or cushion shaped and the colour varies from cream to blue, violet, green, yellow, deep brown, orange or red (see Figure 6). They are often found in dark or semi-dark ecosystems (caves, overhangs, coralligenous substratum). Homoscleromorpha are generally located in shallow waters, but some species have been found below 100 m [66]. They have been perhaps overlooked in deep-sea ecosystems due to their encrusting shape.

The monophyly of Homoscleromorpha has been accepted for many years now [67]–[69], and it was assigned to the rank of a subclass of Demospongiae [69], [1]. However, molecular studies have shown that Homoscleromorpha are not a part of the Demospongiae [64], [70]–[73], and recently, Homoscleromorpha was formally proposed as the fourth class of Porifera [65]. A molecular phylogenetic study based on the internal relationships within Homoscleromorpha has shown that aspiculate and spiculate genera belong to two distinct clades and the families Plakinidae and Oscarellidae, which had been merged in the past have now been restored [74].

Homoscleromorpha is the smallest class of Porifera with two families, 7 genera and 87 species described so far: 16 species of *Oscarella* (Fig. 6A–B), and one *Pseudocorticium* within the family Oscarellidae; 6 species of *Corticium* (Fig. 6E–F), 6 of *Placinolopha*, 28 of *Plakina* (Fig. 6C), 11 of *Plakinastrella*, and 19 of *Plakortis* (Fig. 6B) within the family Plakinidae. Altogether, 40 species have been described in the last 20 years, representing an increase of 42% of the number of Homoscleromorpha. This clade has thus the highest rate of descriptions of new species [66], [75]–[76]. 25% of the species have been described from the Mediterranean Sea 10 of which since 1992. This high level of biodiversity in the Mediterranean Sea is a reflection of special efforts undertaken by a Mediterranean team to find new tools to discriminate between cryptic species. It is predictable that a high diversity of homoscleromorph sponges is present in other regions such as the Caribbean and the Indo-West Pacific.

The Homoscleromorpha are considered too difficult to differentiate at the species level due to lack of diagnostic characters, especially in genera without skeleton *(Oscarella)*, resulting in the perception that many species are cosmopolitan. The high rate of descriptions of new species is linked to genetic studies, which show that morphological variability between sympatric populations is linked to low levels of genetic identity between them [77], [78]. All possible morphological datasets (external features, spicule shapes when present [Fig. 6D–E], anatomy, cytology, microsymbionts) as well as molecular and chemical markers are used as diagnostic characters to discriminate between these species [74], [76], [79]–[83]. The cytological dataset of Homoscleromorpha facilitates discrimination between cryptic aspiculate species of *Oscarella* [76]–[77], [84]–[86], as well as spiculate species of *Plakina* [80]. Muricy [75] emphasized the benefit of inclusion of histological and cytological characters in the taxonomy of other spiculate homoscleromorphs such as *Plakortis*, *Plakinastrella*, *Placinolopha*, and *Corticium*.

#### Calcarea

Calcareous sponges have a mineral skeleton composed entirely of calcium carbonate, consisting of free, rarely linked or cemented, diactine, triactine, tetractine and/or polyactinal spicules, to which can be added a solid basal calcitic skeleton. The aquiferous system ranges in complexity from the most simple (asconoid and syconoid) to a more complex arrangement (leuconoid). The mode of reproduction is viviparous and the larvae are always hollow (blastula) [87].

Calcarea are also called Calcispongiae in the older literature, and more recently in molecular studies. Some authors [88]–[90] propose to use the name Calcispongiae for the Recent representatives to distinguish them from the exclusively fossil Heteractinida (with polyactine spicules).

**Figure 7:**
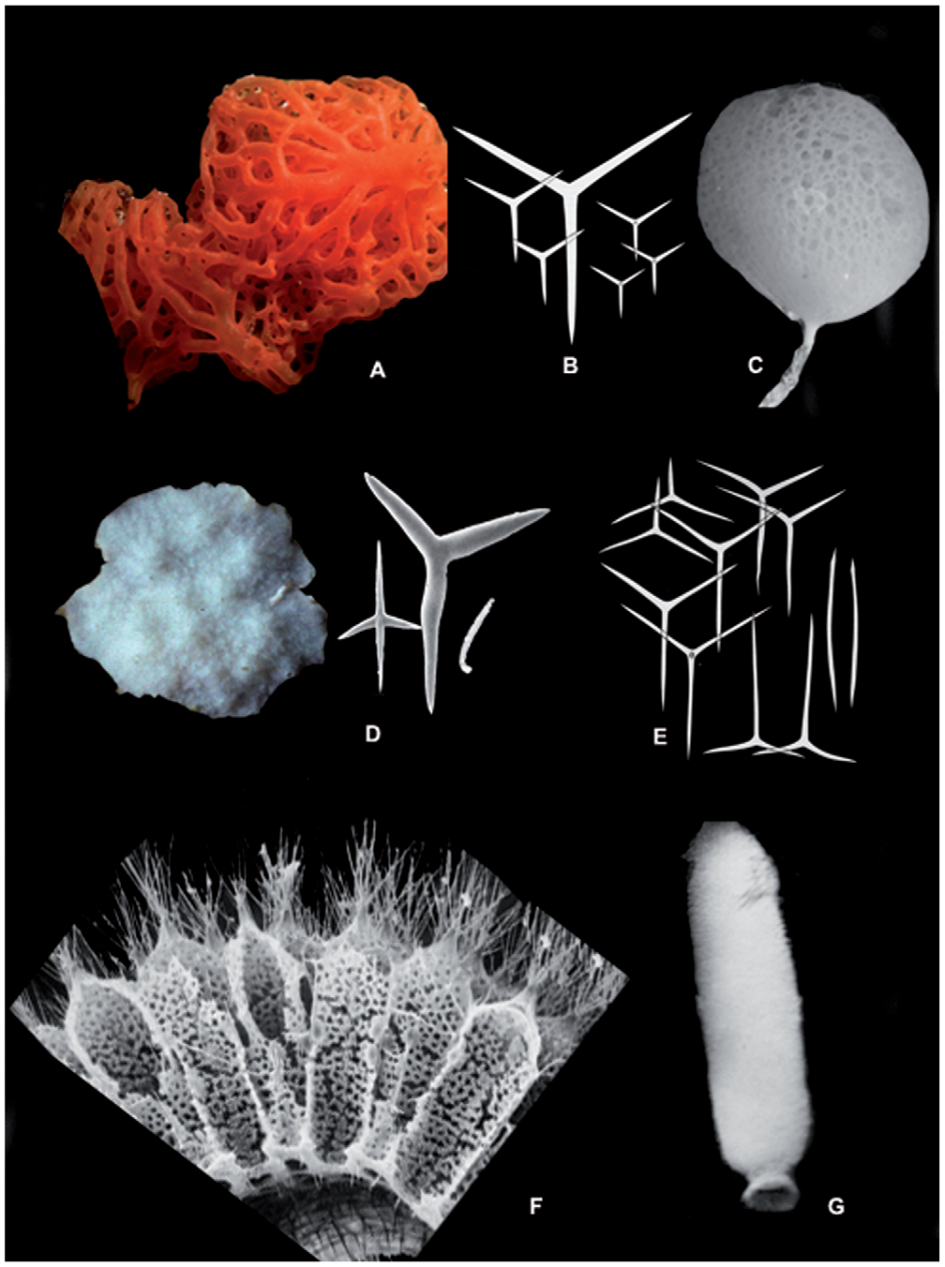
Calcarea diversity. A. *Clathrina rubra* (Calcinea, Clathrinida), NW Mediterranean Sea (photo courtesy Jean Vacelet); B. Calcinean spicules: equiangular and equiradiate triactines (photo courtesy Jean Vacelet); C. *Guancha lacunosa* (Calcinea, Clathrinida), NW Mediterranean Sea; D. *Petrobiona massiliana* (Calcaronea, Lithonida), two specimens from caves, NW Mediterranean Sea. Spicule complement of *P. massiliana*: from left to right pugiole, sagittal triactines, microdiactine (photos courtesy Jean Vacelet); E. Calcaronean spicules: sagittal (inequiangular) triactines and diactines; F. Syconoid aquiferous system from *Sycon ciliatum* (SEM photo, courtesy Louis De Vos, ULB); G. *Sycon ciliatum* (Calcaronea, Leucosolenida), specimen about 10 cm, from the English Channel.

Living calcareous sponges are often delicate with thin coalescent tubes (Fig. 7A, C) or may be urn-shaped (Fig. 7G). Some cave-dwelling species are stony (Fig. 7D). Most of the species are white or cream, but some species may be also red, yellow or pink (Fig. 7A). Calcareous sponges are relatively small, measured in mm or a few cm, however in especially rich temperate estuaries *Sycon ciliatum* can reach more than 50 cm in length and 3 cm in diameter [91]. Pacific coral reefs may also harbor several larger species such as *Leucetta avocado* and *Pericharax heteroraphis*, which may reach 20 cm in height. In most textbooks calcareous sponges are regarded as exclusively shallow-water organisms. However, calcareous sponges are repeatedly collected from bathyal and abyssal zones in the North Atlantic as well as in the Southern Ocean [92]. Knowledge of living calcareous sponges is fragmentary: the total number of described species (ca. 680) represents only about 8% of all described extant sponges. This is partially due to a bias in taxonomic effort and the common perception that calcareous sponges are difficult to identify. More recently, efforts have been made to better understand Calcarea diversity in several poorly studied biogeographical areas, e.g. [93]–[95], and in deep-sea ecosystems [92]. As an example, 67 species of *Clathrina* are now known, with 22 species described since 2000 [94], [96].

The monophyletic origin of calcareous sponges, with their unique morphological feature of monocrystalline calcareous spicules, has never been seriously doubted; molecular phylogenies using the full 18S and partial 28S rDNA sequences confirm with high support the monophyly of the Recent Calcarea [97]–[100].

Currently, the accepted classification is that proposed by Bidder [101] following observations by Minchin [102], and which is based on the position of the nucleus within the choanocytes, the shape of the spicules, the type of larva and the first type of spicule to appear during ontogeny. Bidder's classification [101], based on several independent datasets and recognized by several subsequent authors [103]–[104], was only adopted at the end of the 20^th^ century and validated by the first molecular results [98]–[100]. The two clades recognized within Recent Calcarea are the Calcinea and the Calcaronea. Calcinea has equiangular triactine spicules (Fig. 7B), a basal nucleus in the choanocytes, a flagellum arising independently from the nucleus, a coeloblastula larva, and triactines as the first spicules to appear during ontogenesis. Calcaronea possess inequiangular triactines (Fig. 7E), an apical nucleus in the choanocytes, a flagellum arising from the nucleus, a stomoblastula larva which after eversion (turning inside out) becomes an amphiblastula, and diactines as the first spicules to appear during ontogenesis.

Within Calcinea, 166 species have been allocated to two orders (Clathrinida and Murrayonida). Within Calcaronea, 515 species have been allocated to three orders (Leucosolenida, Lithonida and Baerida). The family Grantiidae (Calcaronea) has the highest biodiversity with 206 species, 138 of which within the genus *Leucandra*.

Congruence between the molecular results and the current classification [87] is not apparent at lower taxonomic levels [88], [96], [98]–[100], necessitating a thorough revision through an integrative approach.

#### Recent developments from molecular phylogenetic studies

Phylogenomics has recently suggested solutions for decades of differing class-level hypotheses on poriferan phylogeny by showing that sponges are monophyletic, and that classes Demospongiae and Hexactinellida form a sister group to classes Calcarea and Homoscleromorpha [72]. Recent molecular data also shed new light on the classification and phylogenetic relationships within Calcarea, Hexactinellida and Homoscleromorpha, as briefly mentioned in their respective contributions, but Demospongiae systematics appears to be demanding major changes.

**Figure 8:**
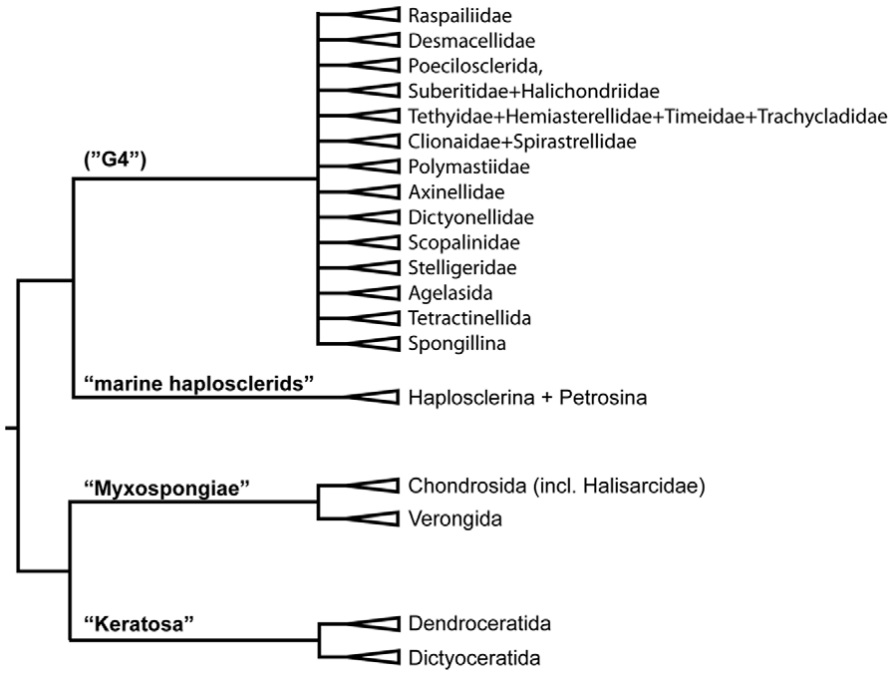
Phylogenetic relationships of higher demosponge taxa as evident from various molecular phylogenies. Sources e.g., [70], [107], [109]. The approximate composition of the “G4” subtaxa is known, but the phylogenetic relationships of these are still to be assessed.

In Demospongiae (Fig. 8), the usage of molecular systematic techniques revealed weaknesses and inconsistencies of the morphology-based classification (for reviews see e.g. [33], [105]–[106]) and demonstrated that morphological characters are of limited use, especially at higher taxonomic levels. Of particular interest are recent studies using mitochondrial and nuclear ribosomal markers, which independently [70], [107] suggest a deep split between (mostly) spiculose, and (mostly) spicule-lacking demosponges. The latter comprises the Keratosa formed by the orders Dictyoceratida [including Verticillitida, see [108]) and Dendroceratida, and the ‘Myxospongiae’ formed by Halisarcida+Chondrosida (which do not fall in distinct orders, see [105]) as sister group to the order Verongida. This implies that sponge orders with predominantly spongin skeletons are not as closely related as previously assumed and the aster-type spicules of chondrosids are not homologous to their hadromerid or tetractinellid counterparts.

In the clade of (mostly) spiculose demosponges, the marine haplosclerid taxa (suborders Haplosclerina and Petrosina) split first. The third haplosclerid suborder, Spongillina (freshwater sponges), forms a clade in a more derived position, leaving Haplosclerida non-monophyletic. The orders Hadromerida, Halichondrida, and Poecilosclerida cannot be recovered monophyletic either, see details in [105] and are subsequently proposed to undergo a re-classification based on molecular results [109]. Molecular data revealed that Raspailiidae and Desmacellidae, which are poecilosclerid families without the characteristic chelae-microscleres, are unrelated to the chelae-bearing Poecilosclerida *sensu stricto* (see details in [105]). Likewise, Halichondrida and its families have repeatedly shown to be non-monophyletic in molecular analyses, with some genera closely related e.g. to Raspailiidae or to the order Agelasida. Halichondrid taxa are also found in new taxon compositions (e.g., the re-defined Dictyonellidae and Axinellidae) or in newly erected families of yet unclear relationships to other taxa (e.g., Scopalinidae) [109]. The nominal family Halichondriidae forms a clade with the hadromerid family Suberitidae. The monophyly of the remaining hadromerid families also cannot be demonstrated. Molecular data suggests a hadromerid clade consisting of Tethyidae, Hemiasterellidae, Timeidae, and Trachycladidae, but a monophyletic relationship to other hadromerid families such as Polymastiidae or the closely related Clionaidae and Spirastrellidae still awaits further support [109]. More distantly, molecular data indicate a potential close relationship of some hadromerid genera and some halichondrid taxa, resulting in a proposed re-erection of the family Stelligeridae [109]. The orders Astrophorida and Spirophorida form a monophyletic group for which the previously employed taxon Tetractinellida can be revived. After inclusion of several ‘lithistid’ families, the monophyly of this group and the apomorphic nature of triaene megascleres is supported by molecular data.

#### The Sponge Barcoding Project

The paucity of complex morphological characters in sponges in combination with a high degree of plasticity, increased chances of homoplasy and cryptic speciation make species identification difficult even for the expert. Molecular tools have recently been employed to attempt to surmount such shortcomings of morphological taxonomy by the usage of DNA signature sequences (DNA-Barcoding) [110]. The Sponge Barcoding Project (www.spongebarcoding.org) [111] has been the first barcoding project for a non-bilaterian metazoan taxon and aims to provide DNA-based identification tools for every poriferan species. Currently the Sponge Barcoding Project builds up a reference database from type material and curated collections from various museums, particularly the Queensland Museum, Brisbane.

### The World Porifera Database

The World Porifera Database (WPD) [10] is an online searchable catalogue of all names of Recent Porifera erected since 1759. The catalogue is part of the World Register of Marine Species (WoRMS [112], available: http://www.marinespecies.org) hosted by the Flanders Marine Institute (VLIZ), Oostende, Belgium. It is an aim of the WPD to be *the* world standard for sponge names and the world portal for internet access to information on Porifera. With its expert team of editors, the WPD acts to stabilize and regulate the use of sponge names in science and society. It serves as a tool for taxonomy by facilitating inventories of taxa, literature references, distributional data, and knowledge gaps. A great advantage over traditionally published inventories is the continuous updating that takes place with each new item of taxonomic information that becomes available in the literature. Currently, the WPD contains approx. 20,000 taxon names of which approx. 8,500 are considered valid (see Table 1).

**Table 1:** Standard record of the World Porifera Database (available: www.marinespecies.org/porifera, accessed 2011 Aug 31) with field names (left column) and content of each field (right column).

Field	Content
**taxon name**	e.g. genus and species combination with authorship and year, or ditto for family and higher taxon names, including unique database number
**Classification**	hierarchical, collapsible higher taxa names to which the taxon belongs
**Status**	accepted or unaccepted, checked or unchecked by taxonomic editor
**Rank**	species, genus etc.
**parent taxon**	first higher taxon
**synonymized taxa**	each linked to its own entry
**child taxon names**	each linked to its own entry
**source reference**	e.g. source of original description, basic source of current classification, additional sources
**Environment**	marine, brackish, freshwater or terrestrial
**fossil range**	Recent only, fossil+Recent, fossil only, unknown
**distribution**	linked to pages containing source references and additional data, including a summary map
**specimen**	link to pages containing type specimen information and source references, additional data on individual specimens
**Links**	buttons linking to other internet resources e.g. Encyclopedia of Life, PESI, Genbank etc.
**Notes**	any additional information or explanations of entries
**Images**	thumbnails linking to photos and other illustrations
**Lsid**	unique species name reference number
**edit history**	who created or changed the entry when
**Tree link and Google link**	links to Taxonomic Tree, Google, Google Scholar, Google Images
**Citation**	requested way of citing the entry

#### Basic data

Table 1 lists the fields and their contents for a standard entry in the World Porifera Database. Most fields are linked to further entries and subsidiary databases. Each entry page contains navigation buttons to various sections of the database (Introduction, Species, Distribution, Checklist, Sources) and contact buttons for editors and database managers.

#### Geographic entries

The WoRMS database architecture provides various geographic resources which can be linked to the taxon entries. Editors can choose between three competing global geographic classification systems: terrestrial, oceanic or ‘alternative’. The first two classifications are nation-oriented (for the oceans the basic system is the Exclusive Economic Zone (EEZ) of countries). Among the alternative classifications are FAO Fishing Areas, Longhurst Provinces and Marine Realms (also known as the Marine Ecoregions of the World (MEOWs), see [30], available: http://www.worldwildlife.org/science/ecoregions/marine/item1266.html). For the World Porifera Database emphasis is based on the MEOW system because it is constructed from animal distribution patterns and is also the most refined, and the only hierarchical system of the existing alternative classifications. From a scientific point of view, this appears to provide a good opportunity to explore distribution patterns of sponges (see below), although depth occurrence cannot be properly documented. Proposals for implementation of Global Open Oceans and Deep Sea-habitats (GOODS) bioregional classification (http://www.ias.unu.edu/resource_centre/ocean%20bioregionalisation.pdf), which accommodates open-ocean and deep-sea distributions, have not yet been honored, and this is anxiously awaited. The WPD editors are also in the process of entering the EEZ occurrences as this may facilitate retrieval of information demanded by nation states.

#### Completeness

Literature on the taxonomy of sponges is scattered over thousands of journals and dozens of books spanning a 250-year period, so any claim of completeness is bound to be false. Nevertheless, thanks to informal card systems and early electronic name lists, a basic catalogue was entered relatively quickly into the WoRMS systems. The Taxonomic Tree at the heart of the WPD was provided by the editorial team of the Systema Porifera [9], so we can rely on this resource for completeness of all taxa down to the level of genus and subgenus. Online sources such as the Biodiversity Heritage Library (available: http://www.biodiversitylibrary.org/), Nomenclator Zoologicus (available: http://uio.mbl.edu/NomenclatorZoologicus/), The Zoological Record Online (http://www.ovid.com/site/catalog/DataBase/200.jsp), and other similar resources allowed quick retrieval of (older) literature records.

All in all, we believe that names of all higher sponge taxa and species names for all extant sponges are virtually completely present in the WPD. This does not imply that all combinations of species names and genus names are incorporated, but original combinations and accepted combinations have been entered to the best of our ability. If a combination cannot be found in the WPD it usually means that it is neither an original nor an accepted combination.

#### Accepted and unaccepted names

Original combinations can be declared unaccepted for two reasons: (1) a published statement of synonymy by one or more taxonomists underbuilt by arguments, (2) an implied synonymy based on the Systema Porifera [9]. An example for the latter reason would be that when a particular genus is considered a junior synonym of another older genus by one of the authors of the Systema Porifera then all species described in the junior genus are automatically transferred to the older genus even though in most cases there is no published statement. The Systema Porifera usually only discusses the type species of genera, leaving the status of the remaining species to subsequent reviewers of the genera. If these species were left in their original combination, the structure of the Taxonomic Tree of the WPD would have been compromised. For largely the same reason, the WPD can only accommodate taxon names following the International Code of Zoological Nomenclature, as rivaling codes are incompatible. Species combinations that do not fall under reasons 1 or 2 are considered accepted for the time being, unless they are known insufficiently to assess their genus affinity, in which case they are declared ‘species inquirenda’.

### Sponge diversity

#### Numbers of taxa tabulated

**Figure 9:**
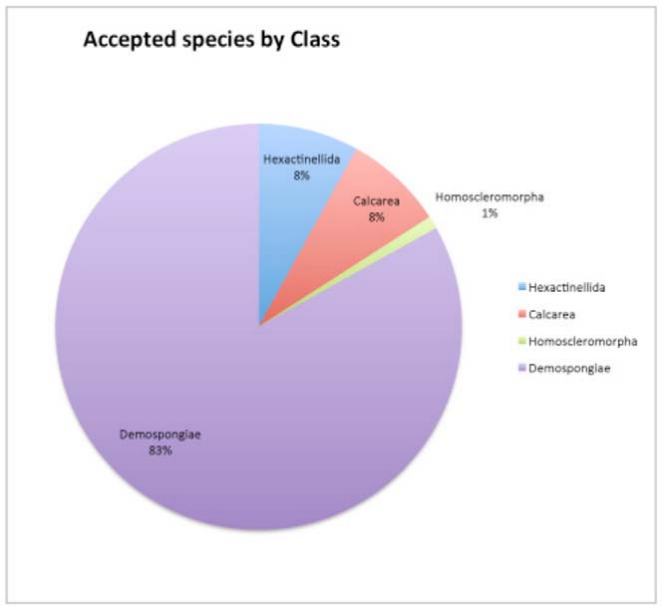
Percentual species diversity of the four classes of sponges. Source: World Porifera Database (available: www.marinespecies.org/porifera, accessed 2011 Aug 31).

Based on the above considerations, Table 2 lists the numbers of WPD entries of species and lower-level combinations (varieties or subspecies) of the four recognized classes. So far (2011 August 31) the number of accepted species of Porifera is 8,553, the vast majority of which (83%) are Demospongiae (Fig. 9). The number of junior synonyms is currently approximately 28% of the number of accepted species. A striking difference in numbers of junior synonyms is observed in the three small classes (respectively 5%, 9% and 3%) as opposed to the Demospongiae (32%). This reflects an overall low scientific effort in the study of these smaller classes: Hexactinellida have attracted few taxonomists over prolonged periods of time, probably due to the perceived difficulty of identification and a lack of taxonomic resource material caused by predominantly deep-sea occurrence. Despite ubiquitous occurrence in many habitats, Calcarea have been neglected as well, possibly because of their small size and apparent uniformity of characters. Homoscleromorpha were only recently separated from Demospongiae [65] and like Calcarea show few classical differentiating features.

**Table 2:** Described species numbers of the four Porifera classes and total number of Porifera species extracted from the World Porifera Database (available: www.marinespecies.org/porifera, accessed 2011 Aug 31).

Taxon	Accepted combination	Unaccepted combination	Unaccepted combination	Total Entries
		junior synonym	genus transfer	
**Demospongiae**	7164	2314	6552	16030
**Hexactinellida**	623	33	427	1083
**Homoscleromorpha**	87	3	108	198
**Calcarea**	681	64	588	1333
**Total species**	8553	2414	7675	18644

Accepted combination: valid species combinations according to the WPD. Unaccepted described species numbers divided in columns ‘junior synonym’ and ‘genus transfer’ combinations of either accepted names or synonyms.

The number of sponge taxa increases steadily at a rate of 35–87 each year, with limited variations over the years, but a striking difference in the number of ‘authors’ for a single new species is apparent over the last century, with an overall single author for each name before the 1980s and a growing number of authors after that. Apparently, species recognition is nowadays a team effort necessitating inclusive authorship.

Taxa equal to or above the (sub-)genus level entered in the WPD number 2004 (see Table 3) overall, approximately half of which (1026) is currently considered accepted, mostly based on conclusions derived from the Systema Porifera. As explained above, higher taxa are under enhanced investigation using molecular sequence data. Rearrangements at all levels are anticipated in the near future.

**Table 3:** Accepted described species numbers (N spp.), accepted numbers of genera (N gen.) and families (N fam.) of higher taxa (suborder and higher) extracted from the World Porifera Database (available: www.marinespecies.org/porifera, accessed 2011 Aug 31).

Class	Subclass	Order	Suborder	N fam.	N gen.	N spp.
**Demospongiae**		Spirophorida		3	11	157
		Astrophorida		6	43	741
		Hadromerida		11	68	750
		Chondrosida		2	5	54
		“Lithistida”		14	51	204
		Poecilosclerida	Microcionina	9	61	874
			Myxillina	11	71	967
			Mycalina	9	46	651
			Latrunculina	1	6	51
		Halichondrida		5	53	689
		Haplosclerida	Haplosclerina	3	27	836
			Petrosina	3	11	248
			Spongillina	8	54	257
		Dictyoceratida		6	41	487
		Dendroceratida		2	8	70
		Verongida		4	10	84
		incertae sedis		n.a.	1	1
**Hexactinellida**	Amphidiscophora	Amphidiscosida		3	12	167
	Hexasterophora	Hexactinosida		9	41	167
		Lyssacinosida		3	55	269
		Aulocalycoida		2	9	12
		Lychniscosida		2	3	8
**Homoscleromorpha**		Homosclerophorida		2	7	87
**Calcarea**	Calcinea	Clathrinida		6	16	164
		Murrayonida		3	3	3
	Calcaronea	Leucosolenida		9	42	477
		Lithonida		2	6	19
		Baerida		3	8	18

#### Numbers of taxa collected but not yet described

There is a large number of ‘unknown’ species: hidden in the many collections worldwide are numerous sponge species awaiting description. Unlike most other marine taxa sponges show dramatic post-collection preservation changes in habit and color, making comparison with living material difficult unless the species has a uniquely recognizable form. Unless good images of living material are available, discovery of new taxa is almost invariably a matter of comparing preserved samples with type material of previously described species. It is not uncommon to discover undescribed sponges in collections that have been preserved for a hundred years or more. The building of a database of *in situ* images in combination with classical imaging and taxonomic descriptions should alleviate the current impediment of post-collection species discovery (see also below).

#### Numbers of taxa expected to be extant

**Figure 10:**
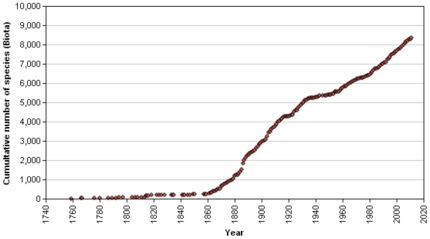
Cumulative increase of sponge species descriptions between 1759 and 2011. Source: World Porifera Database (available: www.marinespecies.org/porifera, accessed 2011 Aug 31).

The cumulative number of described species is increasing at a steady rate (Fig. 10) and there is no indication that it is asymptotic. Regional species accumulation curves may differ as is the case for Australia, where effectively sponge discovery halted after the 1920s and was taken up again only in the last few decades. This caused a dramatically stepped discovery curve with a much steeper-angled increase in recent decades. Following the global curve, it is likely that at the end of the present century the number of known Porifera species will have risen to at least 12,000. New techniques and increased efforts may well accelerate species discovery beyond that. An extra boost in the number of described species may be expected when *a posteriori* morphological studies of previously recognized ‘cryptic’ species, i.e. sponges showing genetic distinctness in the absence of morphological differentiation, are launched in earnest, similar to pioneering studies of e.g. [77], [113]–[116]. A persistent problem, preventing the formal recognition of such cryptic species, is the lack of morphological evidence of such differentiation at the genetic level, e.g. [117]. This is the cause of a widespread reluctance to describe and name these potentially thousands of putatively new species.

#### Numbers of freshwater sponges

Freshwater sponges are united in the suborder Spongillina (Class Demospongiae), numbering approximately 200–250 accepted species. A WPD search produced 257 accepted species, whereas only 219 are acknowledged in [118], the most recent overview of the freshwater sponges. Spongillina are distributed over all continents except Antarctica, and show high endemism with the exception of a few widespread species such as *Spongilla lacustris* and *Ephydatia fluviatilis*. The suborder is divided into six families (and an *incertae sedis* complement), the largest of which, Spongillidae, contains more than half the number of species. There has been some debate over the likelihood of multiple invasions of the freshwater habitat by sponges, so prudency dictates that the issue remains unsolved [118]. However, current knowledge of phylogeny and distribution favours a single Palaeozoic invasion linked evolutionarily to the development of specialized resting stages (called gemmules) found in most freshwater sponges all over the globe.

### Sponge distributions

#### Global distributions

Comprehensive analyses of distribution patterns have been made previously only for the classes Demospongiae [119] and Hexactinellida [120], based on global distributions of all taxa of these classes. The method of these studies was tracing distributions over large pre-conceived areas of endemism. Many more such studies were done in more limited geographic areas, such as Mediterranean-Atlantic [121]–[123], and Antarctica [124]–[125]. More sophisticated attempts at analytical biogeography were invariably more limited in their scope regarding area and/or taxon coverage, e.g. those using biogeographic indices and complicated statistical treatment (Mediterranean-Atlantic areas [126], Australia [127] (see also below), and South Africa [128], or areacladistic analyses (four unrelated genera [129]; suborder Microcionina [130]; genus *Mycale* [131]; 20 selected genera [132]. Panbiogeographic analysis with selected demosponge genera and families was attempted by [133]–[134]. Recently, phylogeographic studies employing various genes at the infraspecific or supraspecific levels were performed with several species complexes in limited geographic areas of the Northeast Atlantic (*Cliona celata* [115], *Phorbas fictitius* [135], deep-water *Hexadella* [116] and *Plocamionid*a [136]) and the Indo-West Pacific (*Leucetta chagosensis* [137]). All these studies were diverse in methodology and taxon content, and it is not possible to arrive at a comprehensive summary at this moment in time.

**Figure 11:**
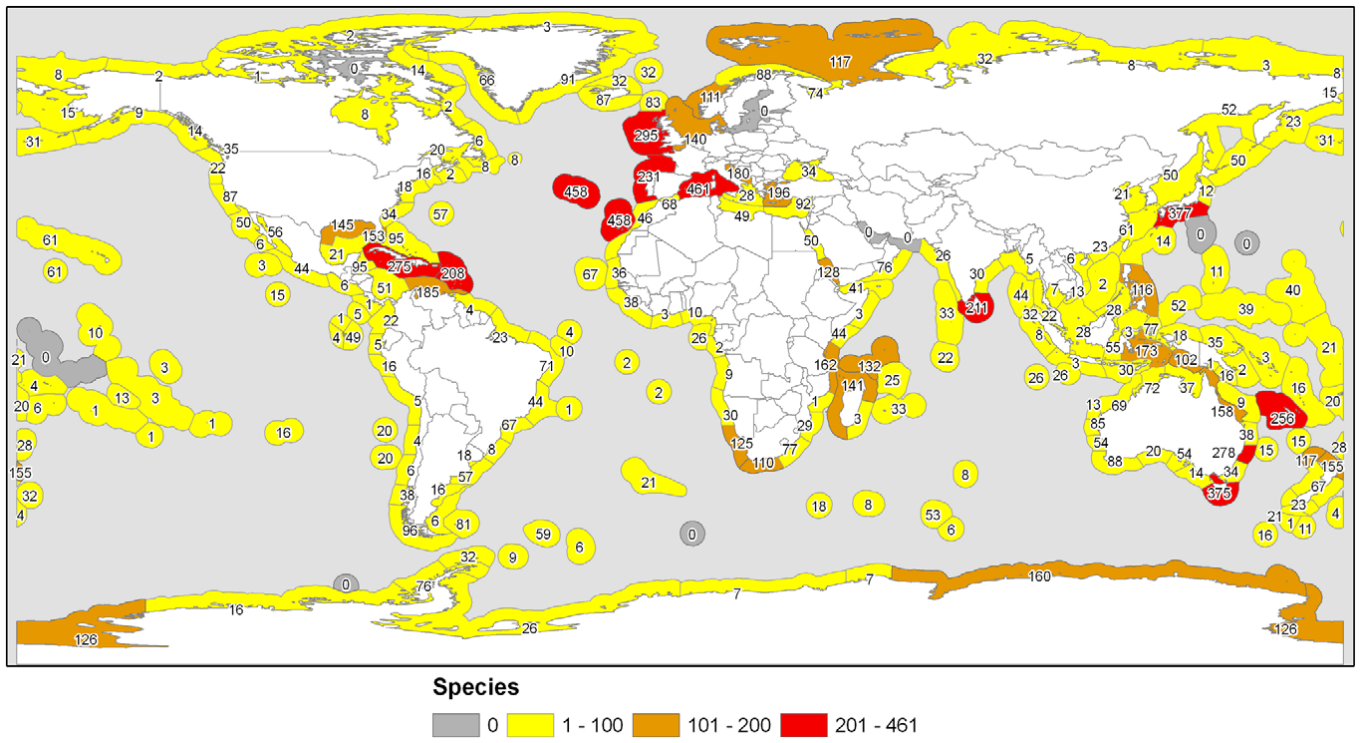
Global diversity of the Porifera. Numbers of sponge species recorded in each of 232 marine ecoregions of the world [30] extracted from the World Porifera Database (available: www.marinespecies.org/porifera, accessed 2011 Aug 31). The type localities and additional confirmed occurrences in neighboring areas of almost all ‘accepted species’ were entered in one or more of the Marine Ecoregions of the World, but many non-original distribution records in the literature are still to be evaluated and entered. Moreover, many sponge taxa are recorded in the literature undetermined and these are not included in the WPD. Thus, the data presented here are to be considered a conservative or ‘minimal’ estimate of the actual distributional data.

Here we will largely confine ourselves to revisit the broader comprehensive approaches made earlier by simply mapping the distributional data from the World Porifera Database into a number of global maps based on the scheme [30] of the Marine Ecoregions of the World (MEOWs), available: http://www.worldwildlife.org/science/ecoregions/marine/item1266.html. Data sets were combined in a geographic information system (GIS) software (ESRI ArcGIS v9.3 [138]), thus numbers of species, genera, and families were plotted into marine Realms, marine Provinces, and MEOWs. From all the maps that we have generated for this study (see the links to individual maps) a clear *collection bias* is evident. This is demonstrated in Fig. 11, which pictures the species content of all MEOWs. Invariably, the most diverse areas appear to be in the Northeast Atlantic, and in more idetail the Mediterranean-Atlantic areas, whereas the tropical coral reef regions, reputedly the most rich areas, come out with lower diversities. We are forced to conclude that current knowledge as laid down in the WPD is likely deficient in showing less than the actual diversity patterns of sponges. Many more areas remain to be explored and many recorded undetermined taxa remain to be named.

Additionally, we present a preliminary biogeographic analysis of the aptness of the MEOW scheme as a tool for representing sponge distributions. In the next section, we will present a clustering of Bray-Curtis indices obtained from comparisons of the sponge contents of all Realms, Provinces and MEOWs. Finally, we will discuss the advantages of a regional approach and briefly review what is known about alien sponge invaders.

#### Marine Realms

At the Realm level species numbers (File S1 part A), disregarding the high number in the North Atlantic for reasons explained above, do reflect partially a pattern that is found in many other marine groups: highest diversity in the Central Indo-Pacific, somewhat lower in the Western Indo-Pacific, lower again in the Tropical Atlantic and lowest in the Eastern Indo-Pacific and Tropical Eastern Pacific. The latter two realms appear severely understudied. Temperate Southern realms probably correctly show highest diversity in Temperate Australasia with its more extended habitats and island groups. The Southern Ocean appears to harbour more species than the Arctic for the same reasons. Genus (File S1 part B) and family (File S1 part C) numbers show similar results.

#### Marine Provinces

Species patterns (File S2 part A) are somewhat surprising, with the Tropical Western Atlantic province as the most diverse province closely followed by the Northeastern Atlantic provinces and the Indo-West Pacific provinces at some distance. Genus (File S2 part B) and family (File S2 part C) patterns are similar, although the differences between the family diversity of circumglobal tropical areas and the Mediterranean and Lusitanian provinces are minimal.

#### Marine Ecoregions

Species patterns (Fig. 11) are complicated and difficult to summarize. MEOWs with high species numbers may be adjacent to very poor ones, often explained by habitat differences (e.g. the 295 species recorded for the Celtic Sea are contrasted by 140 species of the North Sea for reasons of lack of hard substratum in the latter region), but very often also because exploration has been differently intense (e.g. in the South Australian MEOWs). Genus (File S3 part A) and family distributions (File S3 part B) are less extremely different in many MEOWs and probably reflect a more realistic diversity of sponges over the MEOWs more closely than the species distributions.

#### Selected higher taxa patterns: Classes

We provide maps of the species numbers at the Realm (File S4) and the MEOW level (File S5). The demosponge distributions (File S4 part A and File S5 part A) are closely similar to those corresponding to all sponges (see above). Hexactinellida distributions (File S4 part B for Realms and File S5 part B for MEOWs) look surprisingly commonplace, with highest numbers in the West Pacific, but the maps are deceitful by not revealing the predominantly bathyal and abyssal occurrence of these sponges. Calcarea patterns (File S4 part D for Realms and File S5 part D for MEOWs) are obviously biased, with highest numbers in South Australia and Japan and very low numbers in the tropics, reflecting an alarmingly low exploration and description status. Please note that this is the first time a comprehensive map of global Calcarea distributions has been published. Homoscleromorpha is a small group with much of the effort concentrated in the Mediterranean, but the distribution at the Realm and MEOW level (File S4 part C and File S5 part C, respectively) appears to be largely confined to warmer waters.

#### Selected higher taxa patterns: Genera

**Figure 12:**
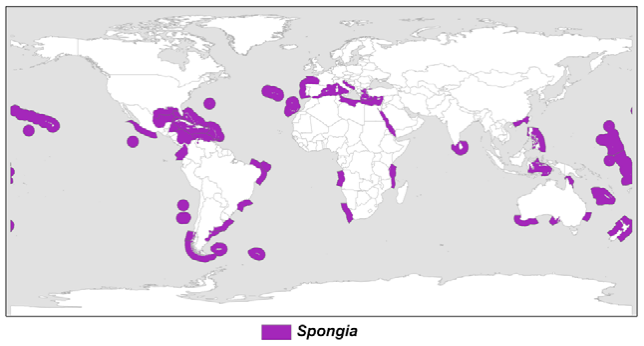
Warm-temperate distribution of the genus *Spongia*. All known species of the genus recorded were entered in the relevant Marine Ecoregions of the World [30], yielding the circumglobal warmer water distribution of this genus. This type of distribution is representative for a large number of sponge genera.

We provide some examples of distinct generic distribution patterns, which were already observed in [119] and later studies. Commonly, genera occur circumglobally in broader or narrower latitudinal zones. Increasingly, patterns that appeared disjunct or restricted at the time have since been found to be much more continuous. Examples of such patterns are: virtually cosmopolitan, e.g. *Tedania* (File S6 part A), warm-temperate, e.g. *Spongia* (Fig. 12), circumtropical, but lacking in the tropical East Pacific and West Africa as found in *Agelas* (File S6 part C), and bipolar/antitropical, e.g. *Iophon* (File S6 part B). Variations on these common distributions are e.g. cosmopolitan with a cold-water bias as in *Myxilla* (S6 part D), bipolar and cosmopolitan deep-sea as in *Asbestopluma* (File S6 part F), and restricted tropical as in *Carteriospongia* (File S6 part E), which is not found outside the Indo-West Pacific. Interestingly, such distribution patterns are not predicted by the hierarchical system [30] of MEOWs, Provinces and Realms: there are no cosmopolitan, bipolar, or circumtropical units distinguished. Obviously, the marine ecoregion subdivision scheme is based on species distributions as they are observed today, lacking biogeographic history. It needs similarity studies to explore such disjunct patterns.

#### Biodiversity analysis: hierarchical clustering of MEOW contents

**Figure 13:**
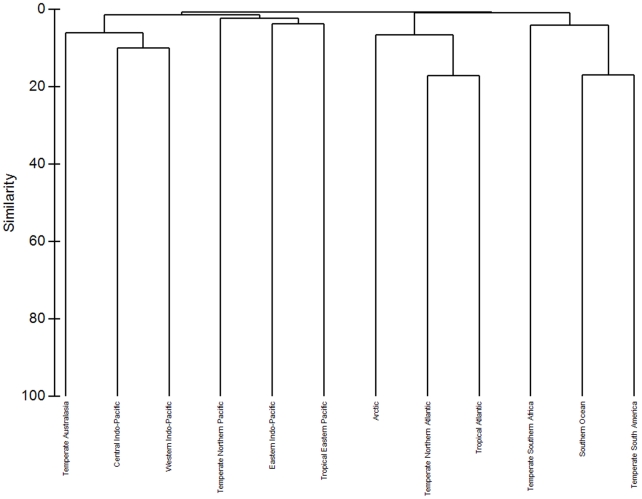
Dendrogram output for hierarchical clustering of 12 Marine Realms. The method used is group-average linking of Bray-Curtis similarities calculated on presence/absence sponge species data. Four assemblage types are identified at various levels of similarity.

Within PRIMER-6 (PRIMER-E package) presence/absence sponge species data were used to perform a hierarchical cluster analysis at Realm, Province (>50 records) and MEOW (>20 records) level. In Fig. 13 the dendrogram is given at Realm level and four assemblage types were identified at various degrees of similarity. The 12 different Realms contained records differing from the lowest number of species present in the Arctic and Temperate Southern Realms (both 310 spp.) and the highest number of sponge species present in the Temperate Northern Atlantic (1664 spp.) and the Central Indo-Pacific (1325 spp.). The different types of assemblage identified represent either the major oceans or a bipolar/antitropical distribution. For instance, the Central Indo-Pacific is most similar to the Western Indo-Pacific together with Temperate Australasia (including Shark Bay and Houtman Abrolhos); the Temperate Northern Atlantic is most similar to the Tropical Atlantic and the Arctic; the Southern Ocean clusters together with Temperate South America and Temperate Southern Africa. The Realms with the lowest number of records cluster together and have a low similarity (Eastern Indo-Pacific and Tropical Eastern Pacific), and these Realms only consist of a few ecoregions of which many have no sponge records at all. They do not only reflect a low exploration status but also the seclusion of their geographical position (e.g. Galapagos, Clipperton, and Polynesia). The endemism of some of the marine ecoregions becomes clearer in the dendogram at the MEOW level (File S7), but in general there are few assemblages at this level that conform to the Provinces distinguished in [30].

The dendogram given at Province level (File S8) also does not clearly represent a nested system where the Provinces are clustered within their Realm. Most Provinces are clustered together adjacent to their closest geographical Province. For instance the Provinces nested within the Western Indo-Pacific Realm are found together with most Provinces of the Central Indo-Pacific Realm (with exception of the Central Indian Ocean Islands and the South China Sea). The similarity of the different identified clusters is very low, reflecting again the low exploration status in many of the Provinces. The Provinces with a high number of records clearly follow the position of the oceans better than provinces with a low number of records.

#### Freshwater sponge distributions

As these were the subject of a recent contribution to the Global Diversity of Freshwater habitats series [118] we will confine ourselves to cite several of the conclusions from that study. Distributions were tabulated in seven classical terrestrial regions (Palaearctic, Nearctic, Neotropical, Afrotropical, Oriental, Australasian and Pacific Oceanic). The most diverse region is the Neotropical region with more than 65 species, closely followed by the Palaearctic regions with around 60 species. Smallest numbers are found on Pacific Oceanic Islands (5 species) and this is also the case for the Caribbean.

At the family and genus level there are some interesting more restricted distribution patterns. The ancient lakes each have distinct endemic species and genera, and the family Lubomirskiidae is restricted to Lake Baikal, the family Metschnikowiidae to the Caspian Sea, and the family Malawispongiidae to the Rift lakes. The family Metaniidae appears restricted to the tropical rainforest belt of all continents, which may be interpreted as a typical Gondwana distribution. This is possibly also the case for the family Potamolepidae, but members of this family are so far not found in Oriental and Australasian forests.

#### Regional data systems and online identification tools

**Figure 14:**
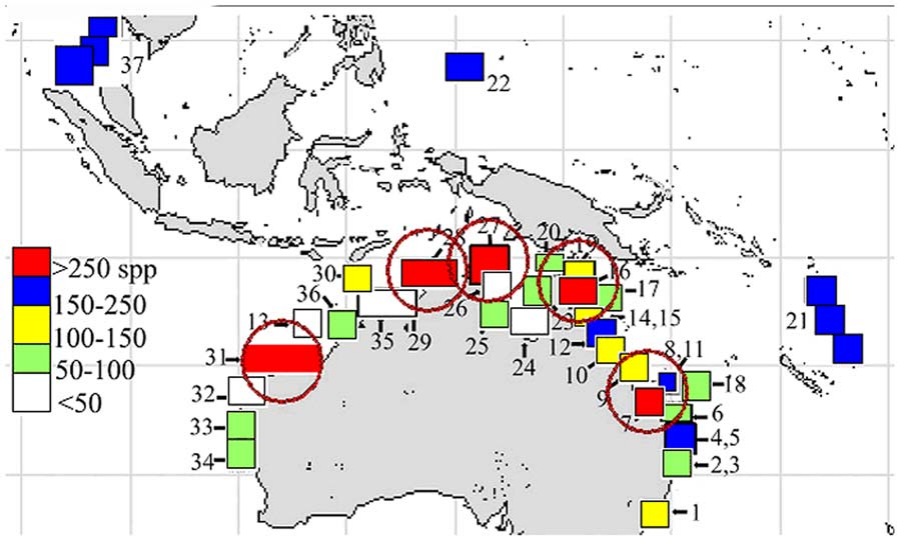
Species richness of regional sponge faunas in western, northern and eastern Australia. Red circles indicate ‘hotspots’ of high species richness (modified from [127]).

Progress of knowledge of global sponge diversity is generated predominantly in many regional efforts, most pre-eminently in the Australian region (Fig. 14). Similar to, but at that time independent of the WoRMS/WPD global effort was an Australian regional inventory of the “known” sponge fauna from the Australian marine territories (amongst the largest in the world, with 6,819,501 km^2^ of seabed jurisdiction, and also the largest in terms of the number of described marine species, 32,900 so far [138]). Since the sponge component of this fauna had largely been untouched since the early 20^th^ century, it also required an attempt to significantly revise this known fauna within a contemporary systematics (ZCA [139]). The initial hardcopy publication listed 1,385 valid species-group names and 338 genus-group names. The subsequent online version (the AFD [140]) currently contains 1,650 species and subspecies in 330 genera and 102 families.

Knowledge of regional marine sponge diversity has expanded considerably over the past two decades in particular, thanks to many new biodiversity discovery initiatives. These range from many small-scale local studies to mammoth voyages over larger continental spatial scales. A few examples are the Great Barrier Reef Seabed Biodiversity project in northeast Australia (GBRSBD [141]), and the Northwest Shelf project in Western Australia [142]. Elsewhere recent expeditions were held and regional guides to sponges were developed of the British Isles [143], North East Atlantic [144], Caribbean [145], Mediterranean [146], New Caledonia [147], Indonesia [148], South Africa [149]–[150], Mariana Islands [151], New Zealand [152]–[153], North East Pacific [154], California [155], Gulf of Mexico [156], Florida [157], and Bahia Brazil [158], to name just a very few. The products from these various initiatives include basic species inventories, but often also extensive databases, websites, CDs and interactive keys. These were derived from an escalated collecting effort over the past two decades driven mainly by a relatively small number of factors. One of these major factors has been the need to know more about the regional inventories of sponge faunas based on the economic potential of sponges for their bioactive compounds as new pharmaceutical products. Biodiscovery for sponges throughout the western Pacific in general, e.g. [159]–[160], and in Australia in particular, e.g [161]–[162], has produced a surge of new sponge collections in the magnitude of several hundreds of thousands of specimens. Another important factor that has accelerated sponge collections is the increasing responsibilities of governments under various agreements within the CBD (http://www.cbd.int/convention) to curb environmental degradation, protect native genetic resources, and improve food security such that marine jurisdictions are increasingly “ground truthing” their seabed for bioregional planning, habitat assessment and conservation purposes. An example of sponge species distributions used in these regional environmental assessments is the rezoning of the Great Barrier Reef Marine Park [163] that included approximately 1,200 sponge OTU's of which even two of the five most prevalent species were new to science, e.g. [164].

To cope with the vast numbers of collected ‘unknowns’ online tools are now available to help fast-track the description and illustration of known and potential new species, including automated taxonomic keys and other initiatives to improve diagnostic capabilities across a range of biota. These tools include EDIT's Scratchpads (www.e-taxonomy.eu), EOL's LifeDesks (www.lifedesks.org), and the ALA-EOL-CBIT (Centre for Biological Information Technology [www.cbit.uq.edu.au] partnership of the IdentifyLife.initiative [www.identifylife.org], amongst others). The Porifera LifeDesks project (porifera.lifedesks.org), currently contains only around 200 species, mostly Caribbean, but is a working component of the Porifera Tree of Life (PorToL, www.portol.org) initiative under development, and is contributing to the global Assembling the Tree of Life project (US NSF funded). In the Australian context the Atlas of Living Australia (ALA, www.ala.org.au) is the most recent development as biodiversity eResearch infrastructure. A further tool in development focused on collaborative work on raw sponge taxonomic data is SpongeMaps wiki (wiki.trin.org.au/bin/viewauth/Marine/Sponges), a new tool that has been developed from the TRIN wiki (wiki.trin.org.au).

#### Invasive species

As in many other marine groups, there are several cases of sponge species known or suspected to have crossed oceanic or terrestrial barriers and showing disjunct distributions. From the 1950s onward, European *Halichondria* species, especially *H. bowerbanki*, have been reported as introduced species in the San Francisco Bay area (available: http://researcharchive.calacademy.org/research/izg/SFBay2K/Halichondria%20bowerbanki.htm). Due to the variability of these sponges and the paucity of distinctive morphological markers the assertion of these being alien species remains inconclusive. An extensively studied case is the Indonesian sponge *Mycale (Mycale) ‘armata’* (identification questioned), which was identified as a potential threat to coral reefs of Hawaii (available: http://hbs.bishopmuseum.org/invasives/reports/mycale.html), following a 1996 invasion of Pearl Harbor. Four other species were identified as ‘unintentionally introduced’ in Hawaiian waters (http://www2.bishopmuseum.org/HBS/invertguide/sponges.htm), the West Indian species *Haliclona caerulea* and *Suberites zeteki*, Philippine *Gelliodes fibrosa*, and Indo-Malayan *Mycale parishi*. These species are not very well known nor do they seem to be reliably identified so we reserve judgement on the source origin. Nevertheless, the monitoring data indicate their recent range extensions. More spectacular is the case of *Celtodoryx ciocalyptoides*, originally described from the Sea of Japan. The species was discovered on the west coast of France from 1996 onwards [165] and described as a new genus and species, *Celtodoryx girardae*, with unknown origin. Very shortly afterwards the sponge was also discovered in the Oosterschelde estuary in the SW part of the Netherlands [166], where it is now one of the more common and conspicuous sponges. Both studies expressed a likely connection with shellfish culture but were unable to provide evidence for this other than that the species was previously unknown from their areas. Henkel & Janussen [167] discovered the likely source populations in the northwest Pacific, and provided convincing proof of the conspecificity of the Asian and European populations. Dutch waters contain several other species not known from elsewhere in adjacent regions and suspected to have been introduced by shellfish transports: *Mycale (Carmia) micracanthoxea*, *Haliclona (Soestella) xena*, and *Sycon scaldiense* [166]. A possible recent introduction from Brazil to the Mediterranean of a calcareous sponge, *Paraleucilla magna*, was reported in [168].

## Discussion

Global diversity patterns of ‘known’ marine sponges very probably reflect sampling bias similar to that which is shown for Ascidiacea [169]. This may be partly explained (a) by our focus on the ‘known’ sponges, i.e. fully described ‘accepted’ species, and the ‘known’ distributions, i.e. vouchered records of ‘known’ species. The scientific literature contains many regional or local species lists with unsubstantiated records of ‘known’ species and undetermined species, and natural history museum collections contain many identified but unpublished specimens that are partly accessible through GBIF and OBIS (iobis.org/mapper, data.gbif.org, 2011-11-05). Although partly to be considered ‘known’ we decided against using these data in view of the mixture of reliable and unreliable identifications inevitably adhering to them. A further explanation for the assumed bias is (b) the lack of reliable identifications of sponges from several of the world's marine habitats, notably all sciophilous and deep-sea habitats, and from several marine regions such as the South East Pacific, the Indian subcontinent, the Arabian and Persian Gulf, tropical West Africa, South East Asia and the Pacific islands. Deep-sea sponge biogeography is still anecdotal. Also, the neglect or lack of effort of the study of major taxa such as the Calcarea and the marine Haplosclerida, respectively 8 and 12% of the total number of species, may have contributed to biased results. Clearly, there is a significant sponge diversity impediment to overcome.

For the next decades, a large amount of sponge specimens and data await treatment. Many of these sponges are already collected and many more are planned to be collected in various regions for biodiscovery and conservation purposes. We have the tools available (e.g. the Systema Porifera classification, the World Porifera Database catalogue, GIS tools, and rapid sequencing) to process these specimens and data, but there is a very significant lag between documenting the specimens, defining these within the Linnaean systematics, and making their distributions widely accessible – the differential being a gap between the “adequately known”, the “poorly known” and the “unknown” in the order of one or more magnitudes. For example, it is estimated that of the >3,000 sponge species collected from North East Australia alone, around 70% are thought to be new to science [170], or cannot be reconciled with any “known”, mostly ancient species concepts as noted above. This also ignores the extra dimension of the quantities of cryptic sibling species hiding amongst alleged widespread morphospecies, e.g. [137], and the tiny, encrusting, parasitic sponge communities that have barely been sampled, and therefore contribute to a potentially even bigger “unknown”. To resolve this using global datasets at the level of realms is at present probably unhelpful, in view of the assumed collection bias, especially when the presently “unknown” (but collected) species are excluded, and without corrections for factors like differential collecting effort and sponge taxonomic research effort between the various regions. Near-future efforts might more productively focus on smaller more manageable regional case studies, whereas the ultimate goal of a global sponge richness assessment is of necessity a distant perspective.

Notwithstanding this, there is great optimism that molecular tools will better define the identities of many of the “known” taxa, and therefore also fast-track the assignment of these vast “unknown” collections to a new or known taxon, e.g. [171]), but much work remains (see Sponge Barcoding Project remarks above).

The MEOW scheme of ecoregions, provinces and realms clearly accommodates only the Recent species distributions and is indeed essentially an ecological instrument. It should be complemented by a higher-taxa scheme of regions, notably for groups of species belonging to the same phylogenetic clade or for genera with unchallenged synapomorphies. Examples we generated here show circumtropical, bipolar, and antitropical distribution patterns, which provide insights in the biogeographic history of taxa and will document faunal changes.

Sponges were initially collected during the halcyon days of curiosity-driven around-the-world expeditions in the 1800s, and in the 1980s they became the focus of the new drive to understand coral reef and temperate marine ecology and invertebrate interactions. Sponges have since escalated in prominence due to their potential value as new sources of pharmaceutical products, transforming our perspective on, and understanding of the biology and biodiversity of these allegedly simple basal metazoans. In conclusion, to our constant amazement, sponges have sustained a high diversity and variety of forms over the entire Phanerozoic Eon, and we continue to find new unprecedented species. We can only imagine the limits of this intriguing group of invertebrates.

## Supporting Information

File S1 **Map showing numbers of sponge species and higher taxa found in each of 12 Marine Realms** [**30**], extracted from the World Porifera Database (available: www.marinespecies.org/porifera, accessed 2011 Aug 31). A. Species numbers, B. Genus numbers, C. Family numbers.
(TIF)

File S2 **Map showing numbers of sponge species and higher taxa found in each of 62 Marine Provinces** [**30**], extracted from the World Porifera Database (available: www.marinespecies.org/porifera, accessed 2011 Aug 31). A. Species numbers, B. Genus numbers, C. Family numbers.
(TIF)

File S3 **Map showing numbers of sponge species and higher taxa found in each of 232 Marine Ecoregions** [**30**], extracted from the World Porifera Database (available: www.marinespecies.org/porifera, accessed 2011 Aug 31). A. Genus numbers, B. Family numbers (for Species numbers see Figure 11).
(TIF)

File S4 **Map showing numbers of species of the four sponge classes found in each of 12 Marine Realms** [**30**], extracted from the World Porifera Database (available: www.marinespecies.org/porifera, accessed 2011 Aug 31). A. Demospongiae, B. Hexactinellida, C. Homoscleromorpha, D. Calcarea.
(TIF)

File S5 **Map showing numbers of species of the four sponge classes found in each of 232 Marine Ecoregions** [**30**], extracted from the World Porifera Database (available: www.marinespecies.org/porifera, accessed 2011 Aug 31). A. Demospongiae, B. Hexactinellida, C. Homoscleromorpha, D. Calcarea.
(TIF)

File S6 **Distribution patterns of representative genera recorded in 232 Marine Ecoregions** [**30**], extracted from the World Porifera Database (available: www.marinespecies.org/porifera, accessed 2011 Aug 31). A. Cosmopolitan distribution of *Tedania*; B. Bipolar distribution of *Iophon*; C. Circumtropic distribution of *Agelas*; D. Antitropical distribution of *Myxilla*; E. Restricted tropical Indo-West Pacific distribution of *Carteriospongia*; F. Deep-sea distribution of *Asbestopluma* (for an example of warm-temperate distribution see Fig. 12 showing the distribution of the genus *Spongia*).
(TIF)

File S7 **Dendrogram output for hierarchical clustering of Marine Ecoregions** [**30**], using group-average linking of Bray-Curtis similarities calculated on presence/absence sponge species data. Of the 232 provinces recognized by [30], those with less than 20 species recorded were omitted, resulting in 132 ecoregions analyzed.
(TIF)

File S8 **Dendrogram output for hierarchical clustering of Marine Provinces** [**30**], using group-average linking of Bray-Curtis similarities calculated on presence absence sponge species data. Of the 62 provinces recognized by [30], those with less than 50 species recorded were omitted, resulting in 44 provinces analyzed.
(TIF)
