# Supplementary figures and images for: Global Diversity of Sponges (Porifera)
Source: PLoS One. 2012 Apr 27;7(4):e35105. doi: 10.1371/journal.pone.0035105 (PMC3338747; doi:10.1371/journal.pone.0035105)

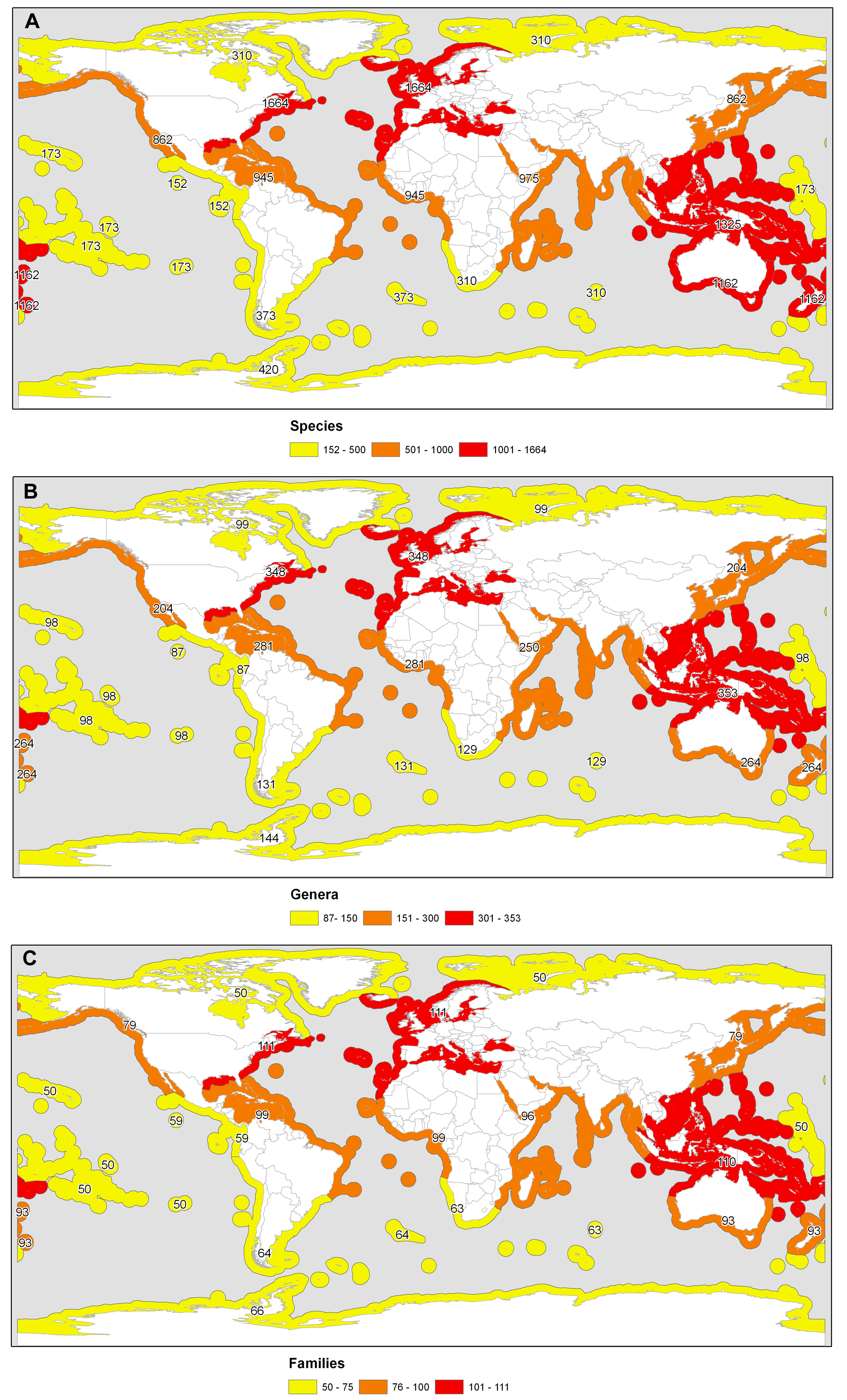

Supplement: File S1 — Map showing numbers of sponge species and higher taxa found in each of 12 Marine Realms [30], extracted from the World Porifera Database (available: www.marinespecies.org/porifera, accessed 2011 Aug 31). A. Species numbers, B. Genus numbers, C. Family numbers. (TIF) [file pone.0035105.s001.tif]

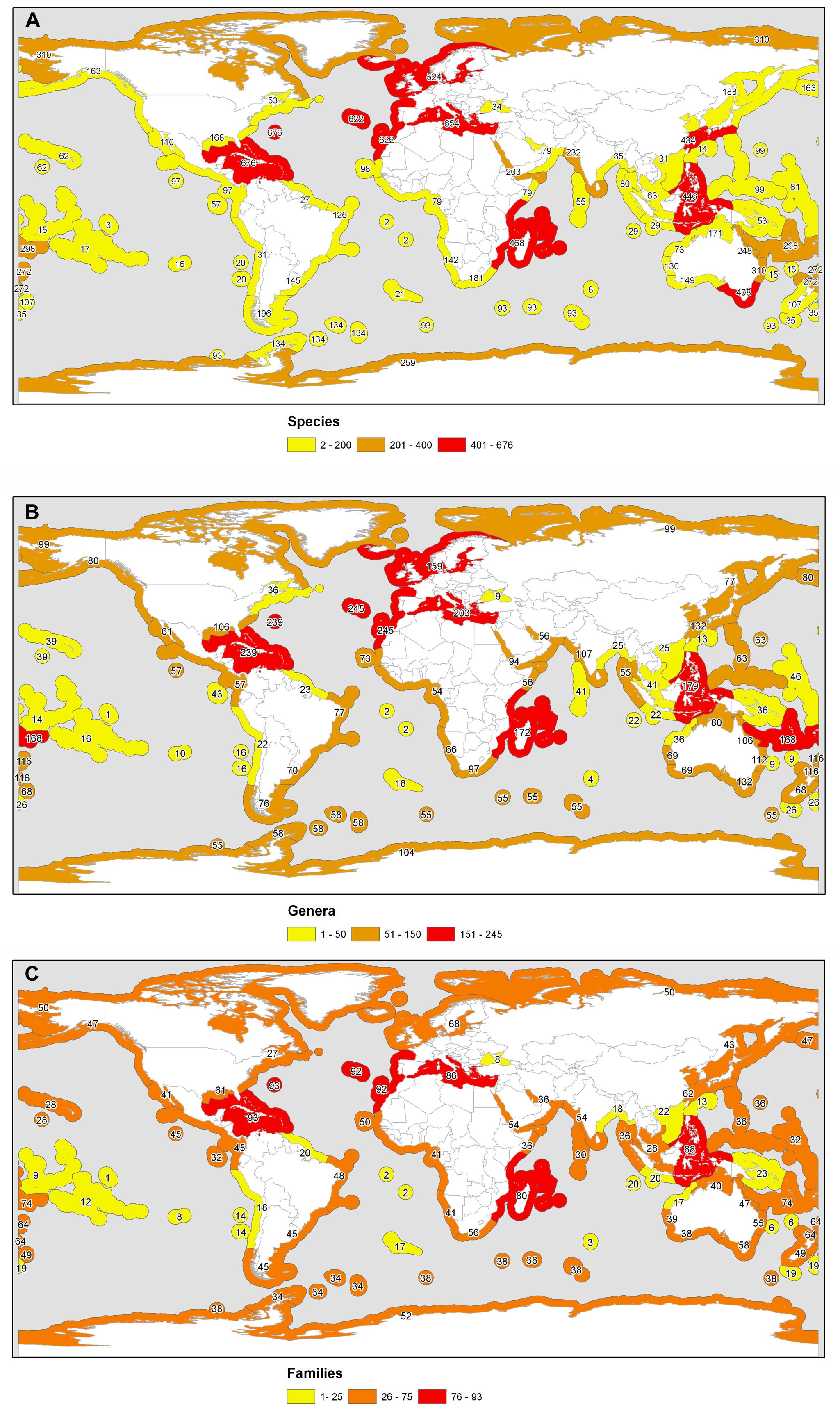

Supplement: File S2 — Map showing numbers of sponge species and higher taxa found in each of 62 Marine Provinces [30], extracted from the World Porifera Database (available: www.marinespecies.org/porifera, accessed 2011 Aug 31). A. Species numbers, B. Genus numbers, C. Family numbers. (TIF) [file pone.0035105.s002.tif]

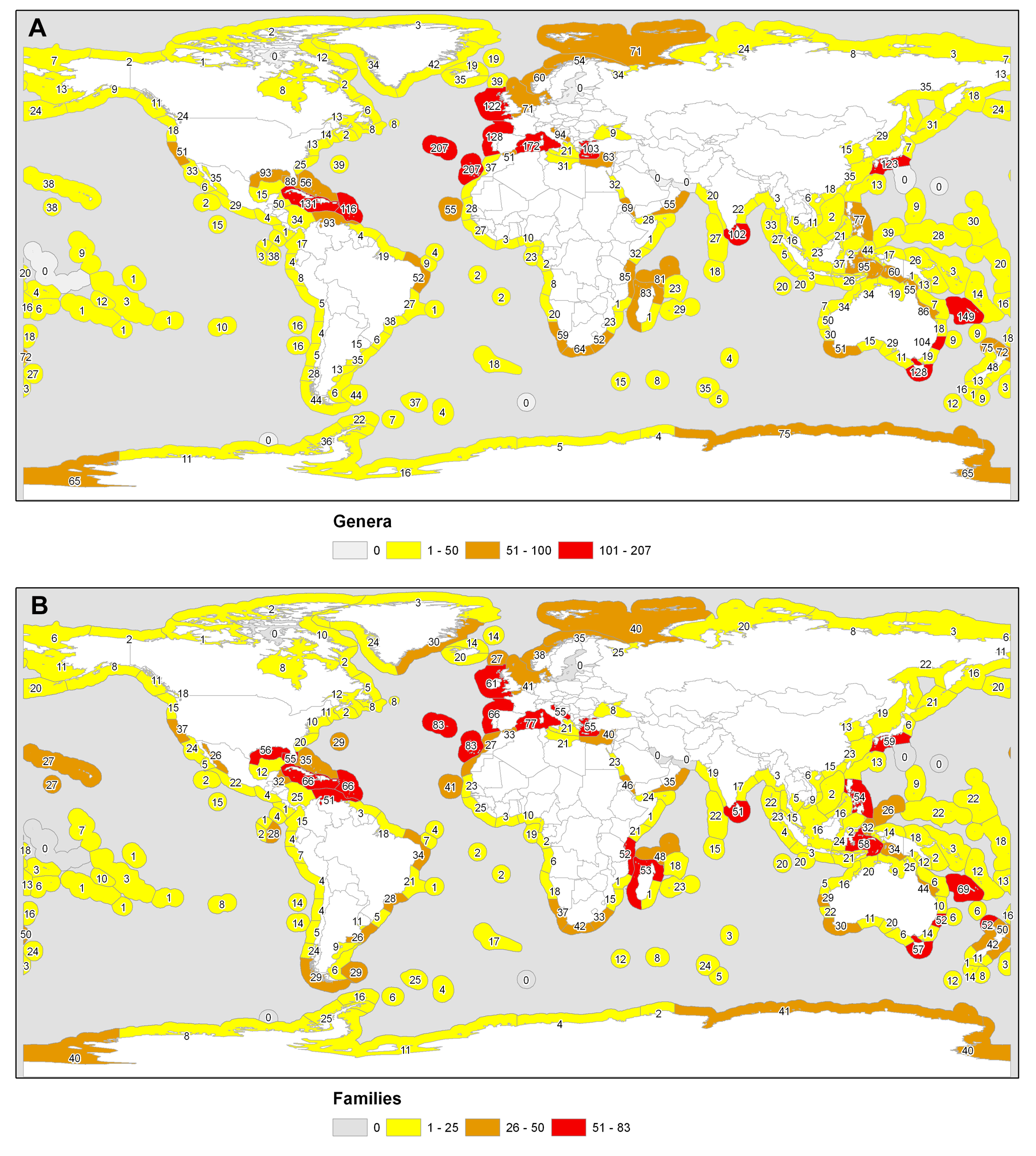

Supplement: File S3 — Map showing numbers of sponge species and higher taxa found in each of 232 Marine Ecoregions [30], extracted from the World Porifera Database (available: www.marinespecies.org/porifera, accessed 2011 Aug 31). A. Genus numbers, B. Family numbers (for Species numbers see Figure 11). (TIF) [file pone.0035105.s003.tif]

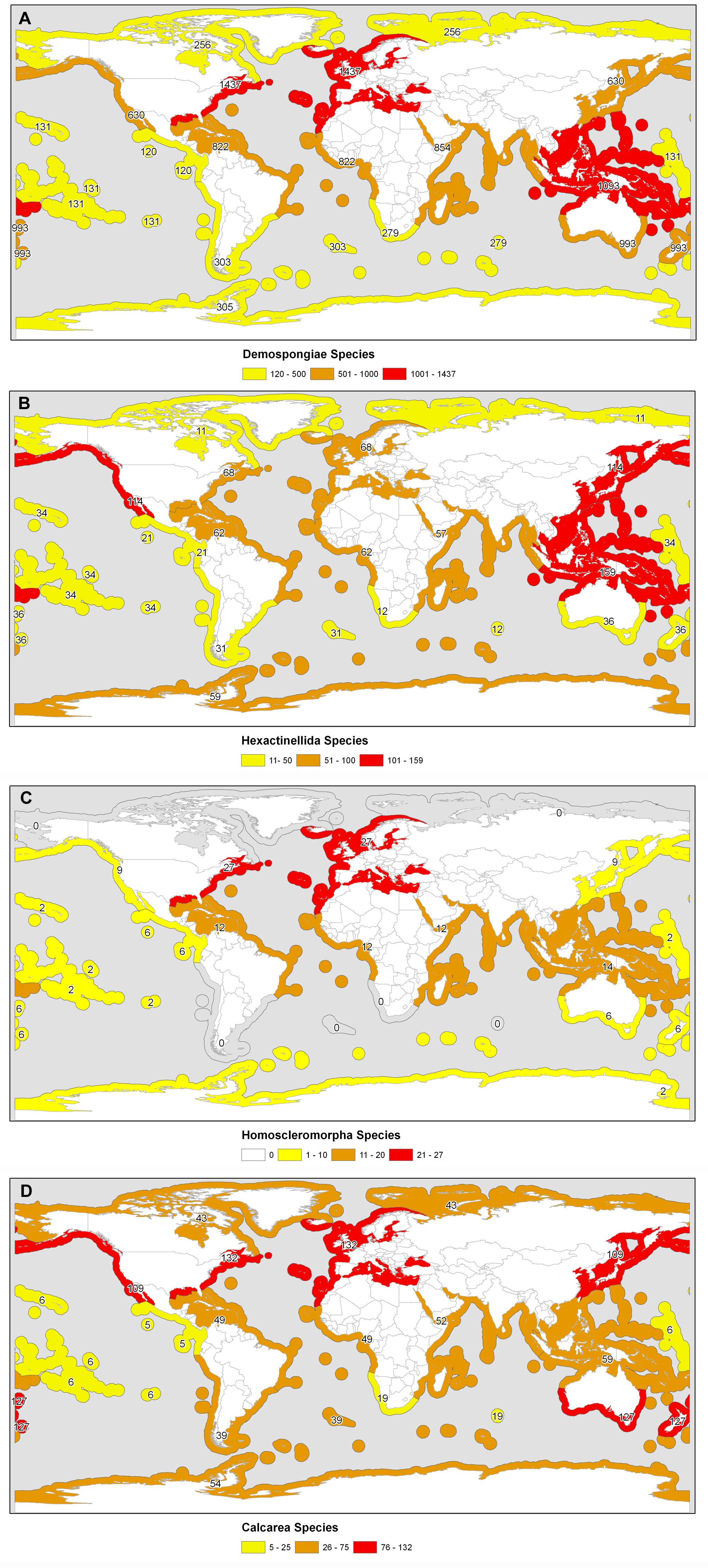

Supplement: File S4 — Map showing numbers of species of the four sponge classes found in each of 12 Marine Realms [30], extracted from the World Porifera Database (available: www.marinespecies.org/porifera, accessed 2011 Aug 31). A. Demospongiae, B. Hexactinellida, C. Homoscleromorpha, D. Calcarea. (TIF) [file pone.0035105.s004.tif]

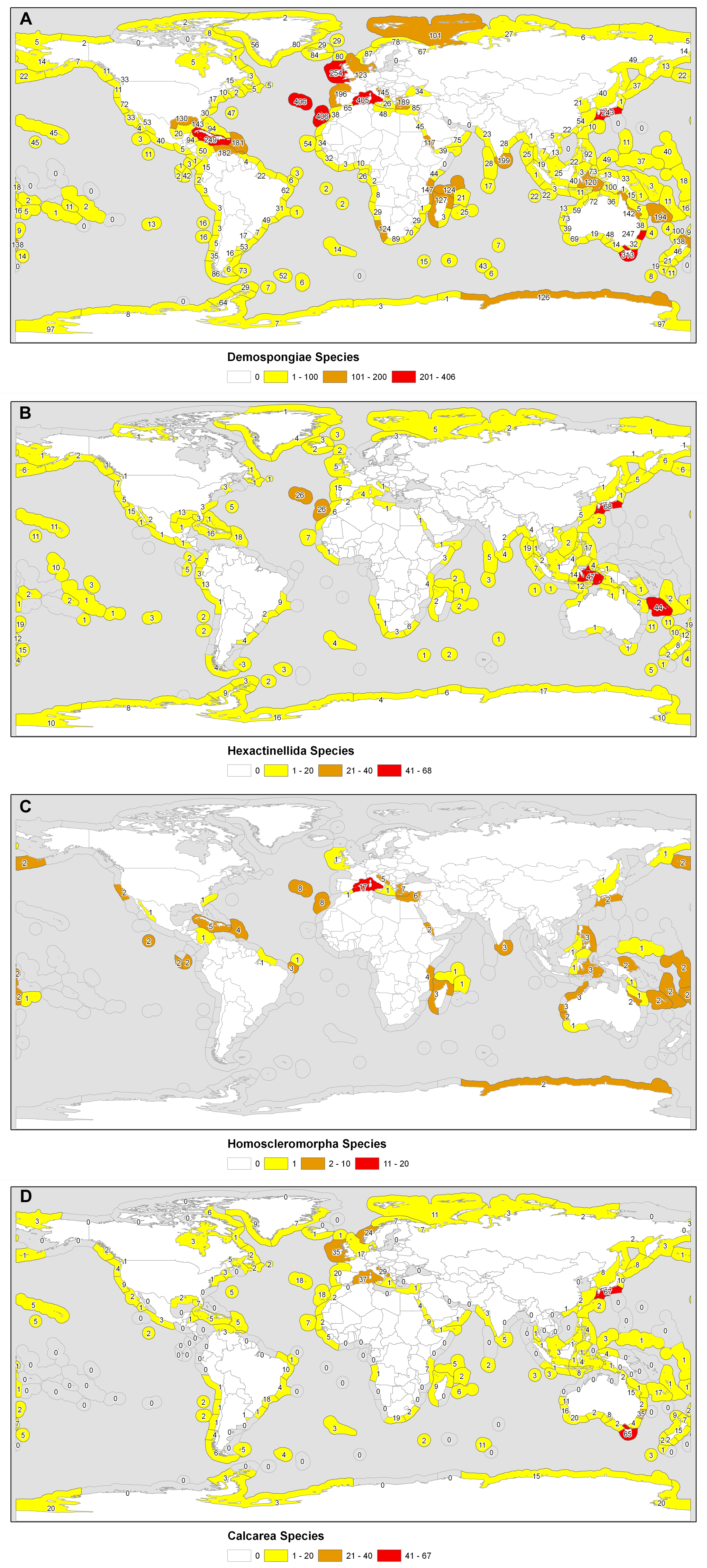

Supplement: File S5 — Map showing numbers of species of the four sponge classes found in each of 232 Marine Ecoregions [30], extracted from the World Porifera Database (available: www.marinespecies.org/porifera, accessed 2011 Aug 31). A. Demospongiae, B. Hexactinellida, C. Homoscleromorpha, D. Calcarea. (TIF) [file pone.0035105.s005.tif]

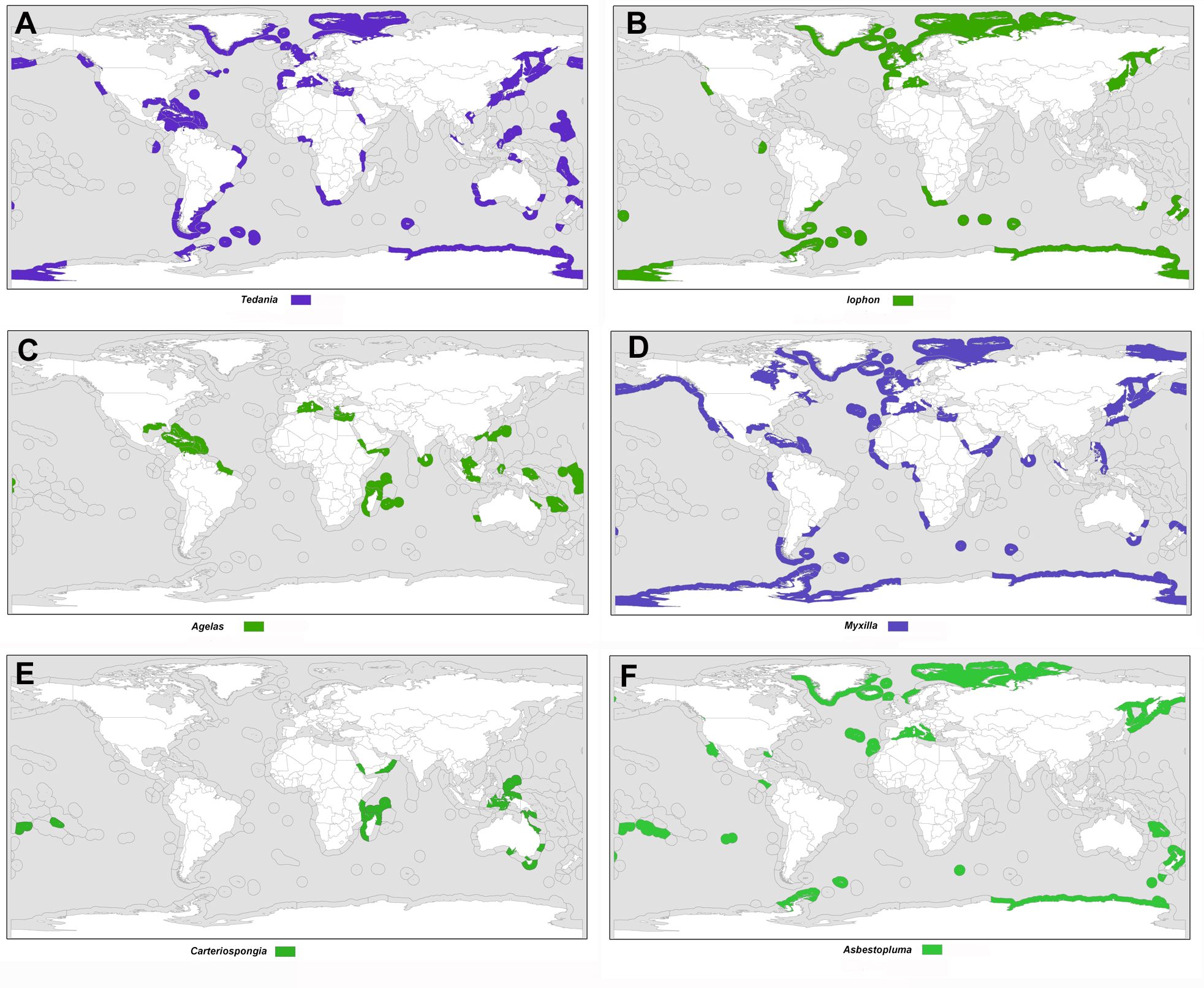

Supplement: File S6 — Distribution patterns of representative genera recorded in 232 Marine Ecoregions [30], extracted from the World Porifera Database (available: www.marinespecies.org/porifera, accessed 2011 Aug 31). A. Cosmopolitan distribution of Tedania; B. Bipolar distribution of Iophon; C. Circumtropic distribution of Agelas; D. Antitropical distribution of Myxilla; E. Restricted tropical Indo-West Pacific distribution of Carteriospongia; F. Deep-sea distribution of Asbestopluma (for an example of warm-temperate distribution see Fig. 12 showing the distribution of the genus Spongia). (TIF) [file pone.0035105.s006.tif]

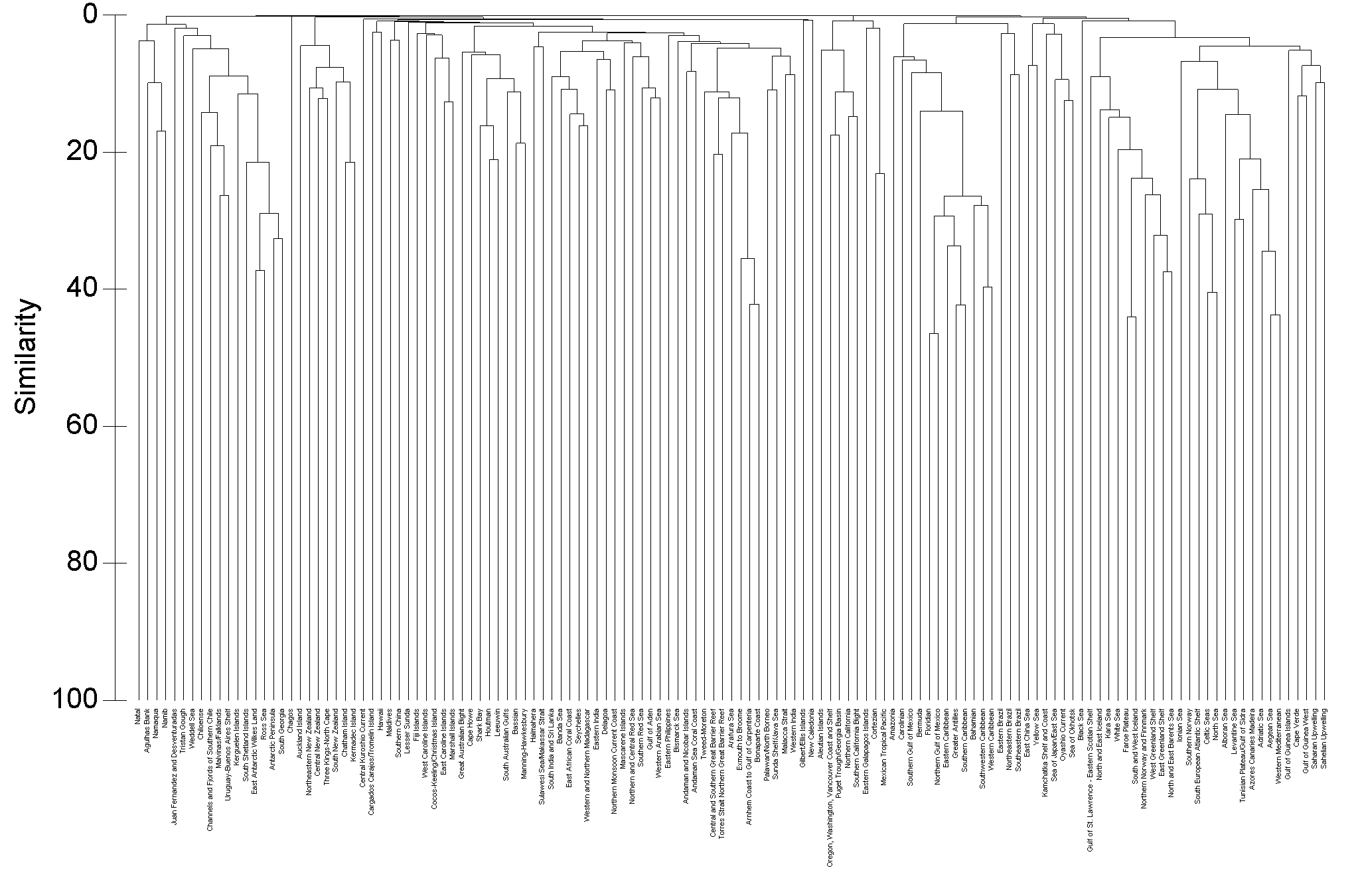

Supplement: File S7 — Dendrogram output for hierarchical clustering of Marine Ecoregions [30], using group-average linking of Bray-Curtis similarities calculated on presence/absence sponge species data. Of the 232 provinces recognized by [30], those with less than 20 species recorded were omitted, resulting in 132 ecoregions analyzed. (TIF) [file pone.0035105.s007.tif]

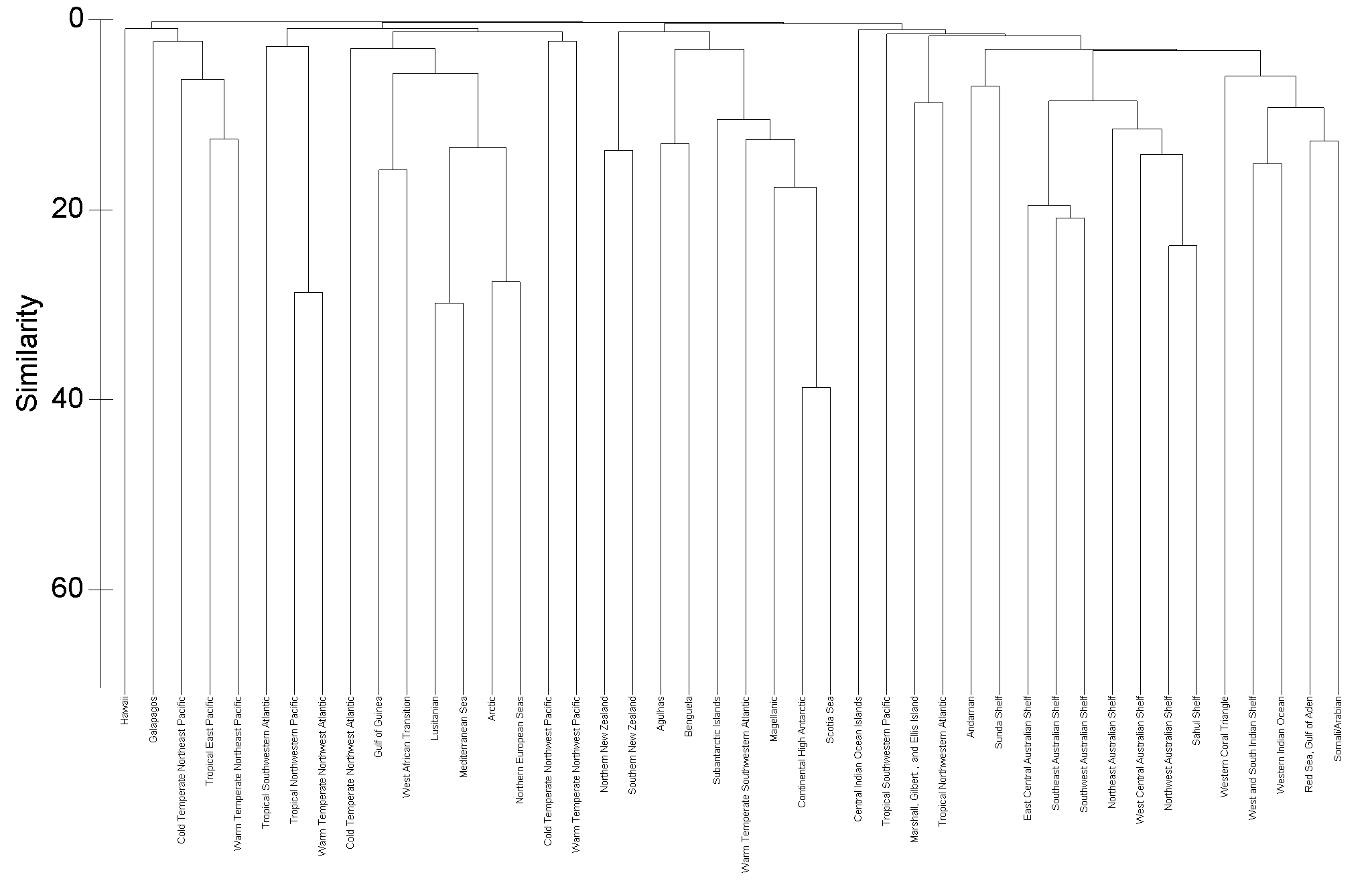

Supplement: File S8 — Dendrogram output for hierarchical clustering of Marine Provinces [30], using group-average linking of Bray-Curtis similarities calculated on presence absence sponge species data. Of the 62 provinces recognized by [30], those with less than 50 species recorded were omitted, resulting in 44 provinces analyzed. (TIF) [file pone.0035105.s008.tif]
